# Sonodynamic and sonomechanical effect on cellular stemness and extracellular physicochemical environment to potentiate chemotherapy

**DOI:** 10.1186/s12951-024-02623-0

**Published:** 2024-06-21

**Authors:** Danli Sheng, Tianzhi Liu, Lang Qian, Jufeng Chen, Yi Wei, Hangrong Chen, Cai Chang

**Affiliations:** 1 Department of Medical Ultrasound, Fudan University Shanghai Cancer Center, Shanghai, 200032 People’s Republic of China; 2 Department of Oncology, Shanghai Medical College, Fudan University, Shanghai, 200032 People’s Republic of China; 3 State Key Laboratory of High Performance Ceramics and Superfine Microstructures, Shanghai Institute of Ceramics, Chinese Academy of Sciences, Shanghai, 200050 People’s Republic of China; 4 School of Biomedical Engineering, Shanghai Jiao Tong University, Shanghai, 200240 People’s Republic of China

**Keywords:** Tumor hypoxia, Deep penetration, Nanodroplet, Ultrasound, Cancer stem-like cell

## Abstract

**Background:**

Hypoxia-activated prodrug (HAP) is a promising candidate for highly tumor-specific chemotherapy. However, the oxygenation heterogeneity and dense extracellular matrix (ECM) of tumor, as well as the potential resistance to chemotherapy, have severely impeded the resulting overall efficacy of HAP.

**Results:**

A HAP potentiating strategy is proposed based on ultrasound responsive nanodroplets (PTP@PLGA), which is composed of protoporphyrin (PpIX), perfluoropropane (PFP) and a typical HAP, tirapazamine (TPZ). The intense vaporization of PFP upon ultrasound irradiation can magnify the sonomechanical effect, which loosens the ECM to promote the penetration of TPZ into the deep hypoxic region. Meanwhile, the PpIX enabled sonodynamic effect can further reduce the oxygen level, thus activating the TPZ in the relatively normoxic region as well. Surprisingly, abovementioned ultrasound effect also results in the downregulation of the stemness of cancer cells, which is highly associated with drug-refractoriness.

**Conclusions:**

This work manifests an ideal example of ultrasound-based nanotechnology for potentiating HAP and also reveals the potential acoustic effect of intervening cancer stem-like cells.

**Graphical Abstract:**

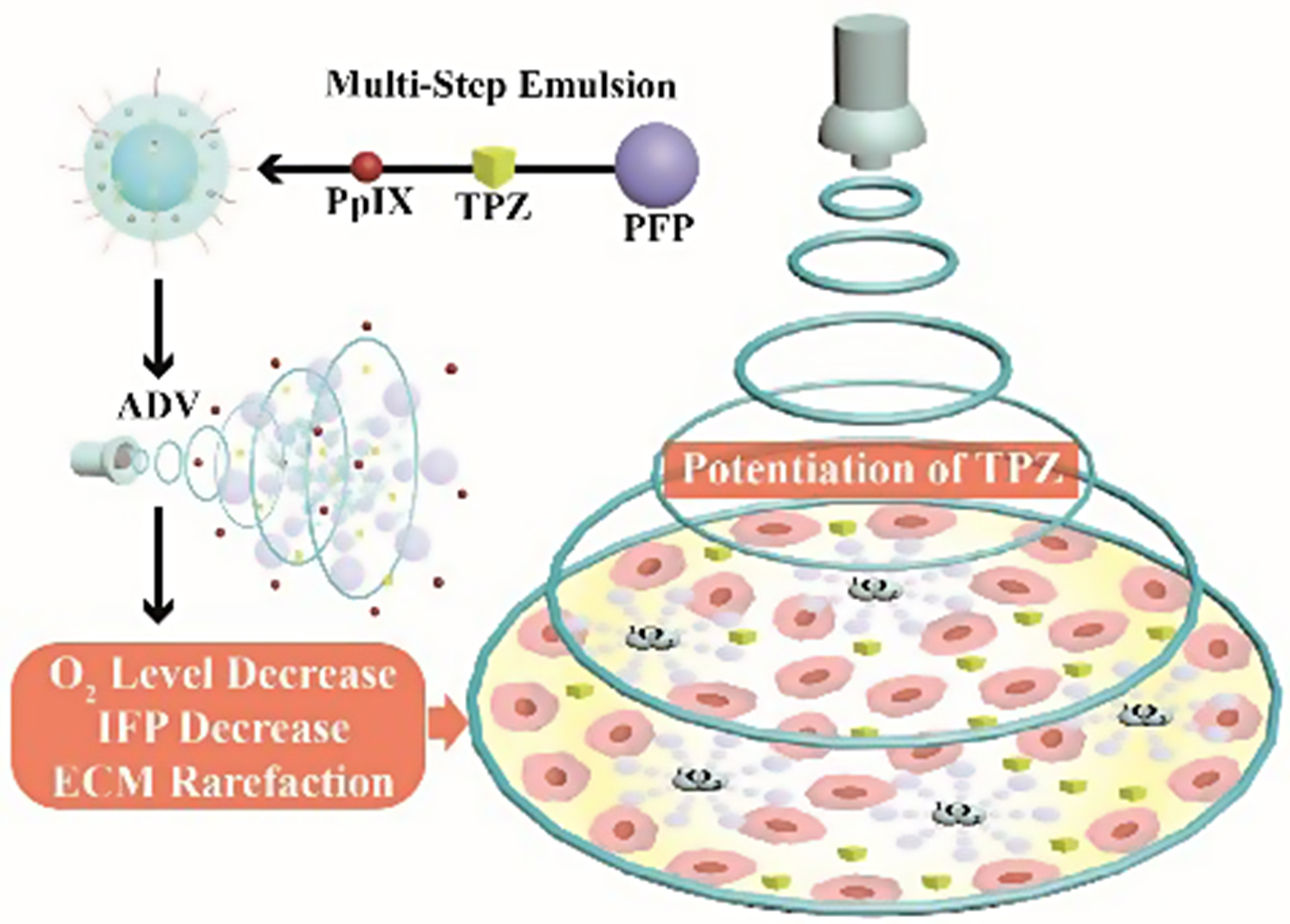

**Supplementary Information:**

The online version contains supplementary material available at 10.1186/s12951-024-02623-0.

## Introduction

Hypoxia is recognized as a common feature of most solid malignant tumors, which has been widely explored as a typical endogenous target for developing tumor-specific therapies in recent years [1, 2]. Among them, hypoxia-targeted chemotherapy has drawn broad attention due to the evolvement of hypoxia-activated prodrug (HAP) [3, 4]. Theoretically, the HAP, e.g., tirapazamine (TPZ), can be reduced to cytotoxic radicals in the hypoxic tumor cells, which exhibits a 300-fold increase in cytotoxic than that in the normoxic region, achieving the hypoxia-dependent chemotherapy [5, 6]. However, as a matter of fact, the oxygen level is relatively normal near the blood vessels, by which the circulated HAP transports into the tumor tissue, while the intratumor hypoxic regions mainly locate at the distance of about 100–180 μm away from blood vessels which is beyond the oxygen diffusion limit [7, 8]. What’s worse, the dense extracellular matrix (ECM), consisting of a highly interconnected network of collagen fibers and reticular fiber, further hinders the intratumor diffusion of HAP [9–11]. Thus, taken together, the majority of HAP can only concentrate in perivascular regions with compromised effectiveness. Last but not the least, in the cell level, a small portion of cancer cells with stem-like phenotype are confirmed to possess high resistance to chemotherapy [12, 13], which are found to reside in the perivascular and hypoxic niches to induce and maintain their stem-like feature [14], thus further impeding the overall efficacy of HAP.

Co-delivery of oxygen-exhausting agent (e.g., photosensitizer, glucose oxidase), using nanoplatform for photodynamic therapy [15, 16] or starvation therapy [17, 18] has been proven to enhance the intratumor efficacy of HAP in the way of promoting hypoxia. In addition, co-delivery of differentiation-inducing agents (e.g., all-trans retinoic acid) with chemotherapeutic drugs has also been developed to render a less stem-like state of cancer cell with reduced drug-resistance for higher chemotherapy efficacy [19, 20]. However, such co-delivery nanocarriers commonly lack deep penetration ability to infiltrate the hypoxic region of tumor. Recently, surface modification technologies have been widely adopted to endow nanomedicine with deep penetration capability [21]. It is reported that surface modification of targeting peptide (e.g., iRGD, TAT) for HAP-loaded nanocarrier can facilitate the intratumor penetration of HAP into hypoxic region for its fully activation [22, 23]. Nevertheless, the design and fabrication of the targeting co-delivery nanoplatform for HAP are still cumbersome, especially for the conjugation of targeting peptides without interfering their performance. More importantly, the inevitable formation of protein corona in the bloodstream could further impair the targeting capability of surface peptides for the nanoplatform [24, 25]. Thus, it is still challenging to enable homogenous delivery and efficacy promotion of HAP simultaneously.

Ultrasound (US), as a mechanical wave, has been commonly utilized as a non-invasive and remote stimulating technique for cancer therapy [26, 27]. Specifically, ultrasound can activate the sonosensitizers to consume oxygen while generating reactive oxygen species (ROS) for sonodynamic therapy or trigger the drug release of ultrasound-responsive nanocarriers [28–30]. Moreover, ultrasound itself can also bring about mechanical or cavitation effect along its propagation path, which can be further magnified by ultrasound-sensitive micro/nano materials, e.g., microbubble, nanobubble and other gas-generating nanomedicine, thus rendering enhanced vasculature permeability and loosen ECM in tumor [31–34]. The acoustic cavitation can not only disrupt the matrix physically, but also produce some reactive oxygen species (ROS), such as singlet oxygen (^1^O_2_) and hydroxyl radical (^.^OH) when irradiated by ultrasound with high intensity as the acoustic cavitation could split water to generate free radicals [35–37]. Notably, it is reported that ultrasound effect can also induce differentiation or reverse chemoresistance of cancer stem cells in vitro and in vivo [38, 39].

**Scheme 1:**
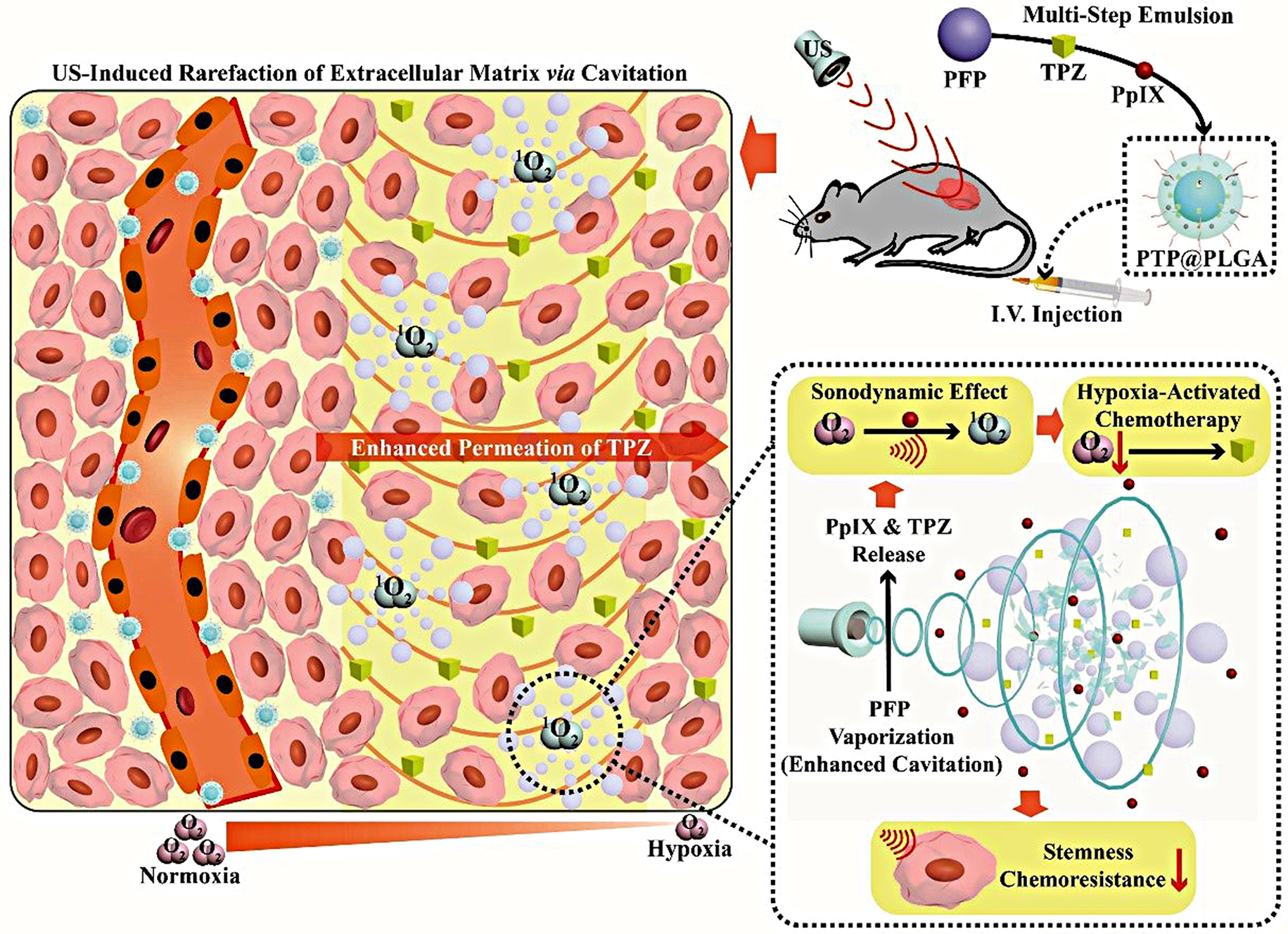
A schematic diagram of the ultrasound-enabled potentiating strategy for HAP based on ultrasound-responsive nanodroplets (PTP@PLGA) *via* the sonodynamic and sonomechanical effect

Herein, an ultrasound-enabled potentiating strategy for HAP is proposed based on ultrasound-responsive nanodroplets (PTP@PLGA). By means of a multi-step emulsion method, the chosen HAP, *i.e.*, tirapazamine (TPZ), perfluoropropane (PFP) and protoporphyrin (PpIX) are co-encapsulated into the poly (lactic-co-glycolic acid) (PLGA) stabilized nanodroplets. As shown in Scheme 1, under the precise ultrasound stimulation at the tumor site, the intratumor accumulated nanodroplets undergo disintegration due to acoustic vaporization of PFP, releasing the loaded TPZ and PpIX. More importantly, the sonomechanical effect (cavitation) is intensively enhanced during the vaporization, which can induce ECM rarefaction in tumor, enabling the increased permeation of TPZ into the highly hypoxic region away from the blood vessels. Concurrently, the sonodynamic effect of PpIX further reduces the oxygen level to activate the TPZ retained in the relatively normoxic region near blood vessels *via* turning the oxygen to cytotoxic singlet oxygen. The overall ultrasound effect can also interfere with the stemness of cancer cells to further reduce their potential chemoresistance. Taken together, the intratumor efficacy of HAP is comprehensively enhanced with an ideal ultrasound-assisted nanotechnology.

## Results and discussion

### Synthesis and characterization of the nanodroplets

**Fig. 1:**
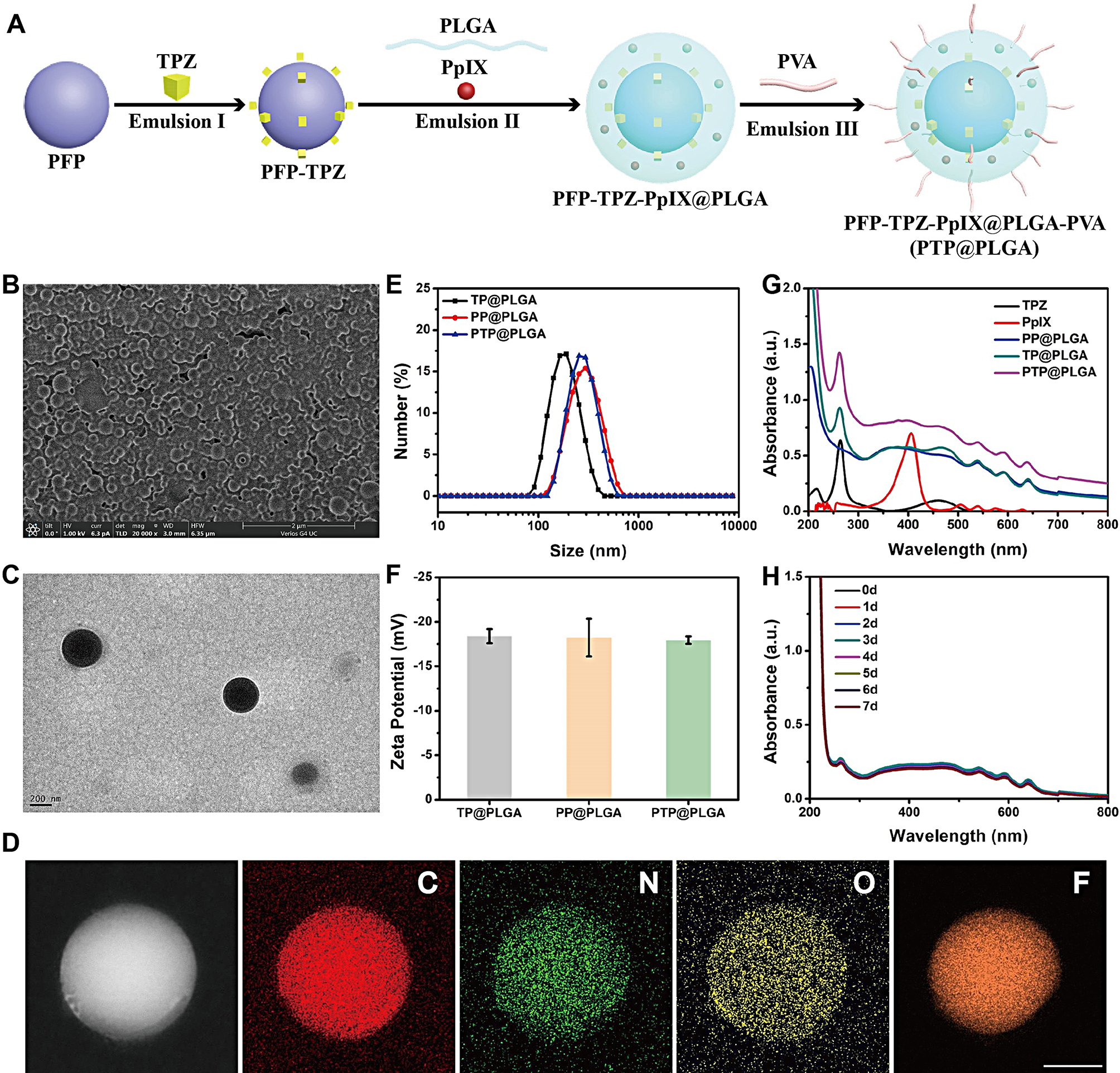
Preparation and characterization of the nanodroplets. (**A**) Synthetic procedures of PTP@PLGA *via* a three-step emulsion method. (**B**) SEM image and (**C**) TEM image of PTP@PLGA. (**D**) Element mappings of PTP@PLGA. Scale bar = 100 nm. (**E**) Dynamic light scattering results of TP@PLGA, PP@PLGA and PTP@PLGA. (**F**) ζ-potential of TP@PLGA, PP@PLGA and PTP@PLGA. (**G**) UV-vis spectra of TPZ, PpIX, PP@PLGA, TP@PLGA and PTP@PLGA. (**H**) UV-vis spectra of PTP@PLGA in the dark for 7 days

As shown in the schematic diagram (Fig. 1A), in order to achieve the co-loading of PpIX and TPZ within the PFP-based nanodroplets, the PTP@PLGA was synthesized *via* a three-step emulsion method. Comparatively, solely without adding PFP or TPZ, TP@PLGA and PP@PLGA were also obtained *via* the same emulsion method, respectively. The obtained PTP@PLGA exhibits a uniform spherical morphology as shown in the SEM image (Fig. 1B). TEM image further reveals a typical core-shell structure of PTP@PLGA, in which the darker contrast in the core may ascribe to the successful encapsulation of the hydrophobic PFP in the nanodroplets (Fig. 1C). And the successful encapsulation of PFP was further confirmed by the element mapping of PTP@PLGA. As shown in Fig. 1D, The C, N, O elements evenly distributed in the whole PTP@PLGA and the F element mainly located in the core of PTP@PLGA, which demonstrated the successful encapsulation of PFP. The dynamic light scattering results show that the average hydrodynamic sizes (AHS) of PP@PLGA and PTP@PLGA are about 318.9 ± 105.2 nm (PDI = 0.325) and 302 ± 88.06 nm (PDI = 0.258) respectively. In comparison, without the PFP core, TP@PLGA has obviously smaller AHS of 178 ± 60.55 nm (PDI = 0.137) (Fig. 1E). Besides, the average diameter of PTP@PLGA maintained a relatively stable nanoscale and showed a slight increase from 322.5 to 418.2 nm without obvious change of appearance within a 5-day duration (Figure S1). And due to the same surface composition (PLGA-PVA shell), the mean zeta potentials of TP@PLGA, PP@PLGA and PTP@PLGA are approximately equal (Fig. 1F). Furthermore, the successful encapsulation of PpIX and TPZ, which displayed the characteristic absorbance peak at 500 nm and 265 nm respectively, was confirmed by the UV-vis spectra for PP@PLGA, TP@PLGA and PTP@PLGA (Fig. 1G). Based on the established calibration (Figure S2 and Figure S3), the loading efficiency and content of TPZ in PTP@PLGA were calculated to be 26.1 ± 1.96% and 4.5 ± 0.3% respectively, which were both lower than those of PpIX (91.5 ± 1.75% and 7.8 ± 0.1%). Moreover, as shown in Fig. 1H, Figure S4 and Figure S5, comparing to that of free PpIX, no obvious absorbance decrease was observed for PTP@PLGA in 7 days whether keeping it in dark condition or not, indicating the better stability of the encapsulated PpIX within PTP@PLGA.

### Concurrent ultrasound-induced vaporization and drug release

**Fig. 2:**
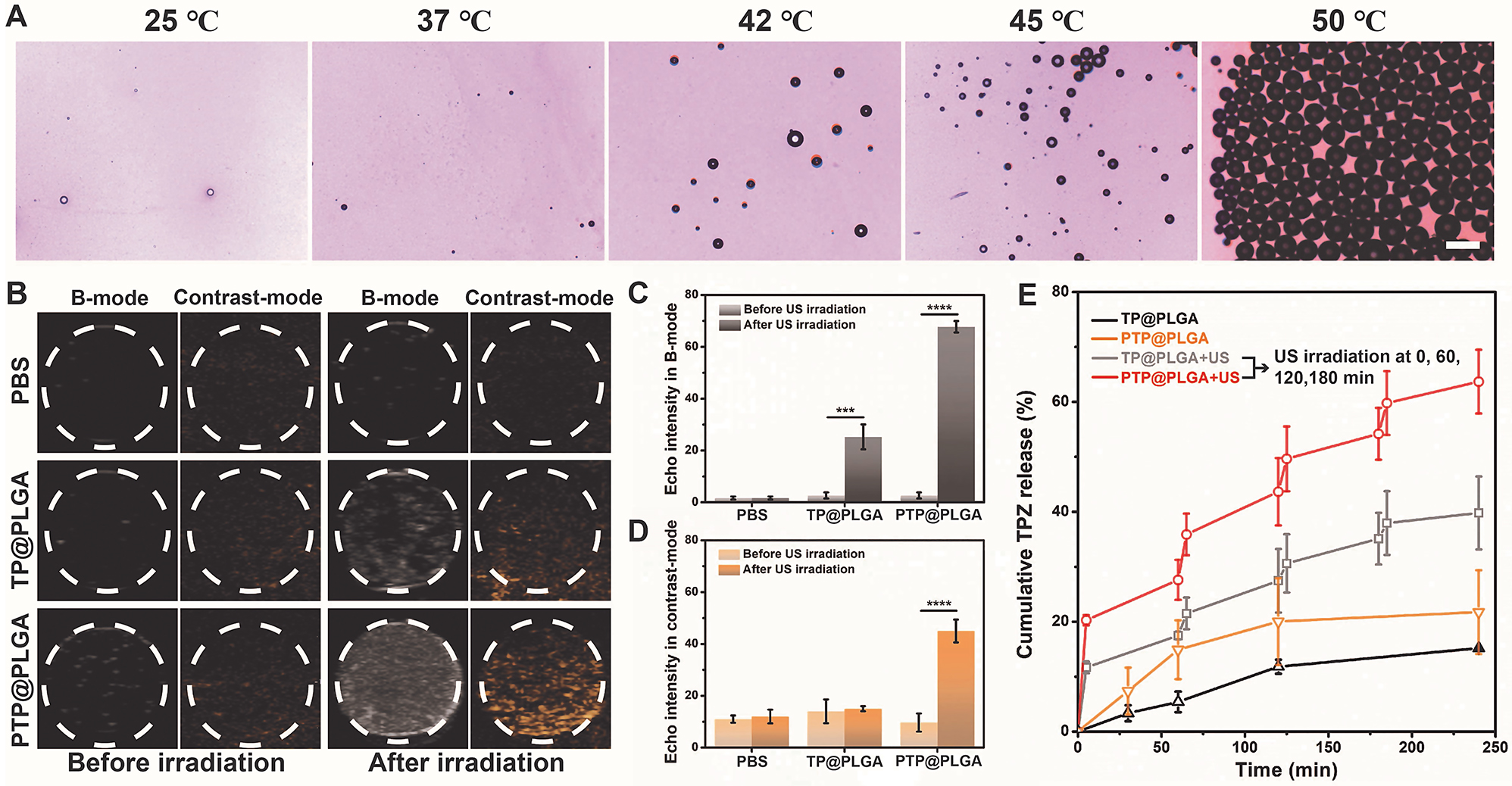
In vitro concurrent ultrasound-induced vaporization and drug release. (**A**) Optical microscope images of PTP@PLGA at different temperatures (Scale bar = 100 μm). (**B**) In vitro B-mode and contrast enhanced ultrasound (CEUS) images and (**C**, **D**) the corresponding echo intensities of nanodroplets (TP@PLGA and PTP@PLGA) before and after ultrasound irradiation. (**E**) TPZ releasing profile of TP@PLGA and PTP@PLGA without/with ultrasound irradiation (1 W/cm^2^, 5 min) at 37 ℃ in PBS

The boiling point of PFP is 29 ℃ under standard pressure, which could be elevated when PFP is emulsified and encapsulated in nanodroplets due to the additional Laplace pressure [40]. For PTP@PLGA, as shown in the optical micrographs (Fig. 2A), only at temperatures above 42 ℃, the encapsulated PFP could vaporize, generating microbubbles with larger diameters. In addition to the thermal stimulation, the vaporization of PFP-based nanodroplets could also be triggered *via* ultrasound due to the applied negative peak pressure, namely acoustic droplet vaporization (ADV). In vitro ultrasound imaging (B-mode and contrast-mode) were further conducted to confirm the ADV process for PTP@PLGA, since the generated microbubbles could act as ultrasound contrast agents. As shown in Fig. 2B, before ultrasound irradiation, both TP@PLGA and PTP@PLGA exhibited no obvious echo changes comparing to PBS due to their nanoscale size. After ultrasound irradiation (1 W/cm^2^ for 30 s), the ultrasound images of PBS remained almost the same. As expected, significantly brighter images were captured in both B-mode and contrast-mode ultrasound imaging for PTP@PLGA, confirming the generation of microbubbles *via* ADV. Comparatively, without the emulsified PFP, TP@PLGA only exhibited slight echo change, which was also confirmed by the quantitative results **(**Fig. 2C and D).

Then, the ADV effect on entrapped TPZ release was further investigated *via* UV-vis spectra. Calculated based on Lambert-Beer law, without ultrasound stimulation, TP@PLGA and PTP@PLGA exhibited only 15.2% and 21.7% release of TPZ in 250 min respectively. As expected, under periodic ultrasound stimulation, the release ratio of TPZ for PTP@PLGA steeply rose up to 61% within 250 min, which was obviously higher than that of TP@PLAG (Fig. 2E). Theoretically, ultrasound itself can accelerate the diffusion rate of drug *via* its thermal or mechanical effect, which has been commonly reported [41–43]. Specifically, for PTP@PLGA, the encapsulated PFP undergoes extreme volume expansion due to ADV, which results in disruption or even fragmentation of the PLGA shell, further facilitating the TPZ release. More importantly, the PFP microbubbles generated *via* ADV are believed to oscillate or even collapse under ultrasound stimulation simultaneously, leading to dramatic cavitation effect, such as microstreaming, shockwave and microjet, which can in turn amplify the thermal/mechanical effect of ultrasound for drug release [44–46].

### Sonodynamic effect induced oxygen consumption

**Fig. 3:**
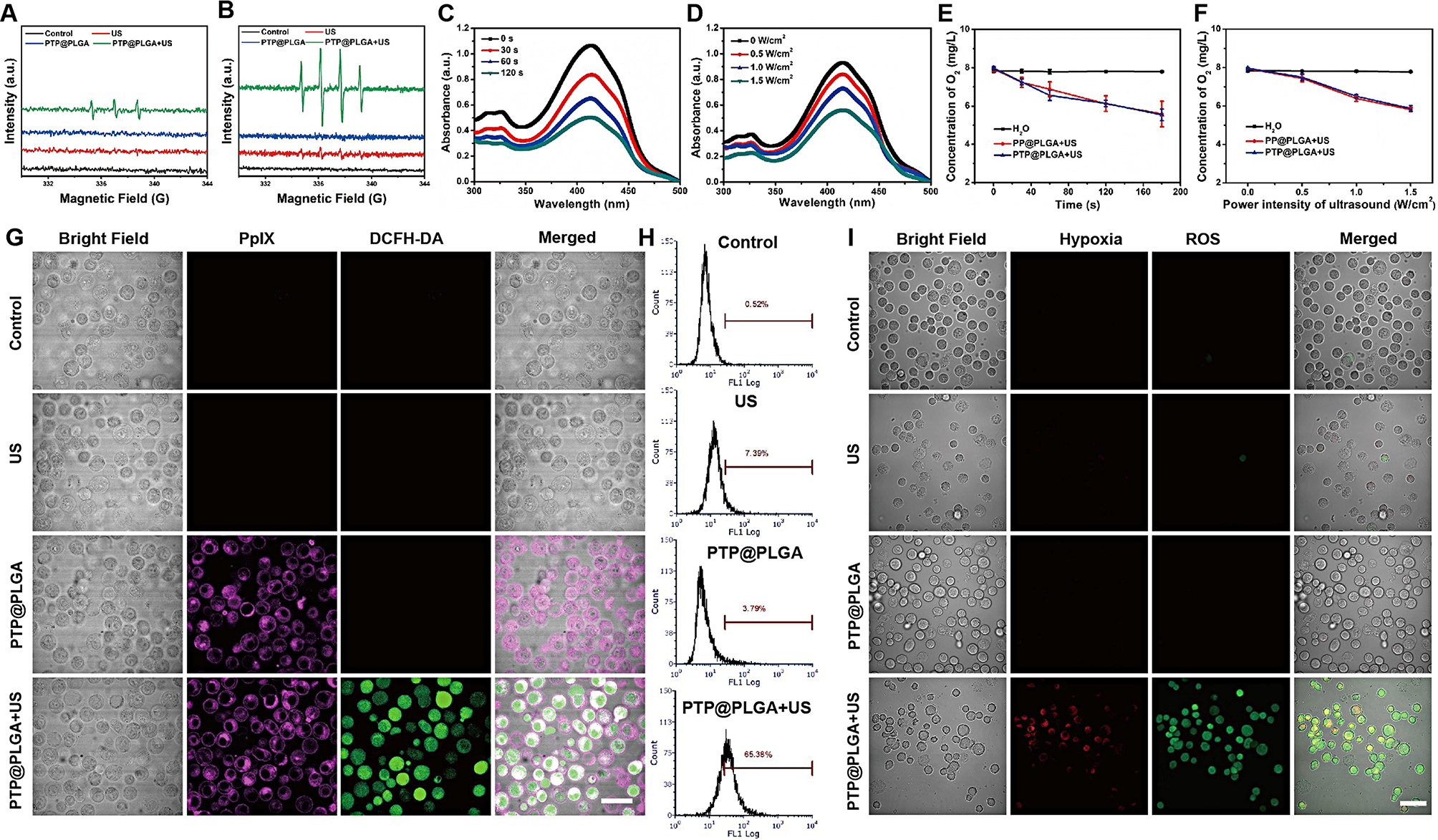
Sonodynamic effect and the consequent oxygen consumption. ESR spectra of (**A**) ^1^O_2_ and (**B**) ^.^OH generation in control, US, PTP@PLGA and PTP@PLGA + US group. The absorption spectra of DPBF incubated with PTP@PLGA under ultrasound irradiation with (**C**) different durations (1 W/cm^2^ for 30, 60 and 120 s) or with (**D**) different power intensities (0.5, 1 and 1.5 W/cm^2^ for 60 s). The change of oxygen concentration in PP@PLGA and PTP@PLGA under ultrasound irradiation with (**E**) different durations (1 W/cm^2^ for 30, 60, 120 and 180 s) or with (**F**) different power intensities (0.5, 1 and 1.5 W/cm^2^ for 60 s). (**G**) CLSM images of DCFH-DA stained 4T1 cells with different treatments (control, US, PTP@PLGA, PTP@PLGA + US). Scale bar = 40 μm. (**H**) The corresponding flow cytometry quantitative analysis of ROS generation in 4T1 cells with different treatments. (**I**). CLSM images of 4T1 cells stained by Hypoxia/Oxidative Stress Detection probe under different treatments (control, US, PTP@PLGA, PTP@PLGA + US). Scale bar = 40 μm

To assess the in vitro sonodynamic effect of PTP@PLGA, the ROS species: singlet oxygen (^1^O_2_) and hydroxyl radical (^.^OH) were firstly evaluated by electron spin resonance (ESR). 2,2,6,6-tetramethypiperidine (TEMP) was used as trapping agent for ^1^O_2_ detection and 5,5-Dimethyl-1-pyrroline N-oxide (DMPO) for ^.^OH detection. As shown in Fig. 3A, three peaks with the same intensity were manifested in the coexistence of PTP@PLGA and US, which suggested that PTP@PLGA could produce ^1^O_2_ triggered by US. Additionally, the capability of PTP@PLGA to generate ^.^OH was also assessed. As shown in Fig. 3B, both the US and PTP@PLGA + US groups displayed the representative ^.^OH signals with an intensity ration of 1:2:2:1. Then the ^1^O_2_ production was further evaluated by using DPBF as trapping reagent, which can react with ^1^O_2_ to form an endoperoxide, showing a decrease at 410 nm in the UV-vis spectrum. As shown in Fig. 3C and D, when incubated with PTP@PLGA under ultrasound irradiation, the characteristic absorption peak at 410 nm of DPBF decayed gradually with the prolonging of duration and increasing of power intensity, showing a typical time and intensity dependent manner. Thus, for PTP@PLGA, in addition to ADV, the applied ultrasound can also activate O_2_, yielding ^1^O_2_ *via* PpIX sensitization. With the same content of PpIX, PP@PLGA and TP@PLGA (Figure S6) exhibited the similar phenomenon under ultrasound irradiation. And no significant difference was found among the above three PpIX-loaded nanocarriers, regarding the DPBF oxidation experiment *via* sonodynamic effect. Meanwhile, during ultrasound irradiation, the oxygen level was also measured *via* a portable dissolved oxygen meter. Due to the efficient sonodynamic effect of PTP@PLGA, the dissolved oxygen could be effectively converted to ^1^O_2_. Thus, the concentration of the dissolved oxygen decreased under ultrasound irradiation, exhibiting a similar time and power dependent manner as shown in Fig. 3E and F. With the same content of PpIX, TP@PLGA showed negligible difference on the change of oxygen concentration in comparison to PTP@PLGA (Figure S7). Thus, it is believed that the PpIX mediated sonodynamic effect can form hypoxic environment for the further activation of TPZ *via* oxygen depletion.

Furthermore, intracellular ROS generation *via* sonodynamic effect was evaluated using DCFH-DA as the ROS indicating agent, which could be converted to dichlorofluorescein with green fluorescence in the presence of ^1^O_2_. As show in the confocal laser scanning microscope (CLSM) images (Fig. 3G), DCFH-DA stained 4T1 cells were divided into 4 groups (control, US, PTP@PLGA, PTP@PLGA + US) for different treatments, in which only the PTP@PLGA + US group exhibited obvious green fluorescence covering the entire cells. The corresponding quantitative analysis *via* flow cytometry further confirmed that the PTP@PLGA + US group yielded the strongest green fluorescence among all the treated groups (Fig. 3H). With the same content of PpIX, the similar results were also found for both PP@PLGA and TP@PLGA (Figure S8). And the SDT-induced ROS generation and exacerbated hypoxia was further evaluated by Hypoxia/Oxidative Stress Detection probe. As presented in Fig. 3I, the 4T1 cells in the control, US and PTP@PLGA groups showed no obvious fluorescence signal. While intensive red (hypoxia) and green fluorescence (ROS) was observed in the PTP@PLGA + US group due to the ROS generation and SDT-induced hypoxia. Thus, as expected, the PpIX-entrapped nanocarriers could be endocytosed in the 4T1 cells, rendering the intracellular ^1^O_2_ generation under ultrasound irradiation.

### Synergetic therapy in cell level

**Fig. 4:**
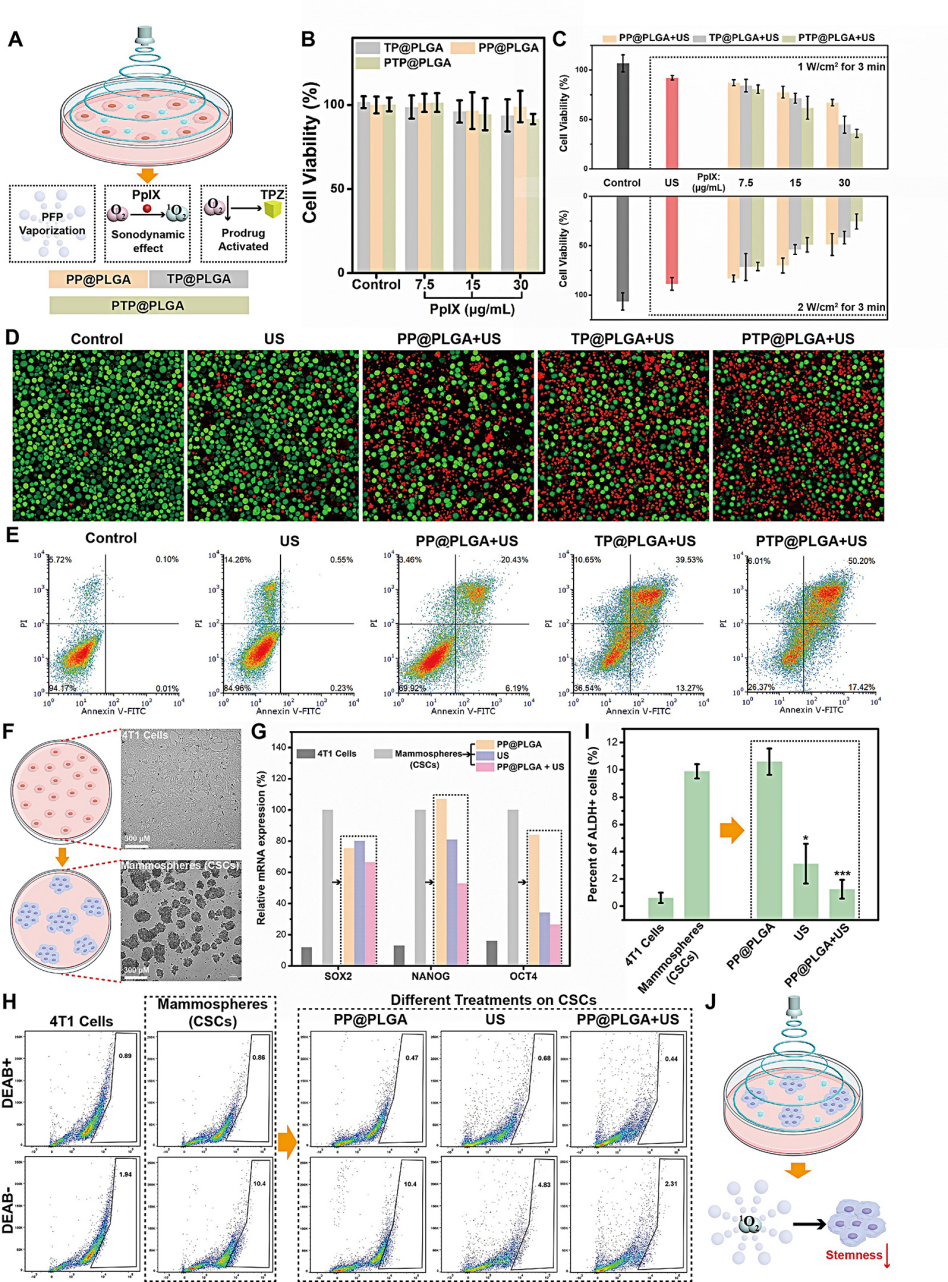
Synergetic therapy in cell level and in vitro ultrasound effect on cancer stem-like cells. (**A**) Schematic illustration of proposed mechanism of different nanodrops under ultrasound stimulation in the cell level. (**B**) Cytotoxicity of TP@PLGA, PP@PLGA and PTP@PLGA with different concentration of PpIX. (**C**) Cell level therapeutic evaluation of different treating groups with two different US power output (1 W/cm^2^ for 3 min and 2 W/cm^2^ for 3 min). (**D**) Confocal fluorescence imaging of 4T1 cells stained with calcein-AM and PI after different treatments. US power output: 1 W/ cm^2^ for 3 min. Scale bar = 70 μm. (**E**) Flow cytometric analysis of annexin V-FITC/PI-stained 4T1 cells after different treatments. US power output: 1 W/ cm^2^ for 3 min. (**F**) The schematic illustration and optical microscope images of the adherent 4T1 cells and the suspended mammospheres (cancer stem-like cells) obtained *via* a serum-free culture of 4T1 cells. (**G**) Expression of stemness-associated genes (SOX2, NANOG, OCT4) in 4T1 cells and mammospheres after different treatments. (**H**) Flow cytometric analysis of the ALDH + proportion in 4T1 cells and mammospheres after different treatments and (**I**) the corresponding quantitative results and statistical comparisons (*n* = 3). (**J**) Schematic illustration of the ultrasound effect for intervention of stemness of CSCs

As designed and shown in Fig. 4A, PP@PLGA, TP@PLGA and PTP@PLGA possessed diverse ultrasound-responsive behaviors, which were further evaluated and compared on therapeutic efficacy for 4T1 cells to reveal the proposed synergetic effect of PTP@PLGA.

As shown in Fig. 4B and Figure S9, without US irradiation, TP@PLGA, PP@PLGA and PTP@PLGA nanodroplets with varied concentrations of PpIX and TPZ all exhibit negligible cytotoxicity to 4T1 cells and 3T3 fibroblast cells at normoxic condition, indicating their excellent biocompatibility. Furthermore, the therapeutic effect of PTP@PLGA under US irradiation was also evaluated in the cell level. As shown in Fig. 4C, under two power output of US (1 W/cm^2^ and 2 W/cm^2^, 3 min), all PP@PLGA, TP@PLGA and PTP@PLGA show significant cytotoxicity, in which, as expected, the PTP@PLGA induces highest cytotoxicity. Thus, it can be inferred that for PTP@PLGA, in addition to the ^1^O_2_ generated *via* sonodynamic effect, the simultaneous oxygen consumption can further activate the hypoxia-sensitive TPZ, resulting in synergistic therapeutic effect, which is superior to PP@PLGA with sonodynamic effect alone. Meanwhile, the cytotoxicity induced by TP@PLGA + US is also lower than that by PTP@PLGA + US since less TPZ could be released from TP@PLGA nanodroplets without the effect of PFP vaporization. Furthermore, the therapeutic outcome was also directly observed *via* CLSM, where the dead cells were stained with PI (red fluorescence) and the living cells were stained with calcein-AM (green fluorescence). As shown in Fig. 4D, red fluorescence was found in the CLSM images of multiple groups, in which the PTP@PLGA + US (1 W/cm^2^ for 3 min) treated group exhibited the most significant red fluorescence, indicating the massive cell death under such treatment due to the synergistic therapeutic effect. The flow cytometric assay was also conducted to investigate such ultrasound-mediated therapeutic effect. As shown in Fig. 4E, compared to other treated groups (control, US), PP@PLGA, TP@PLGA and PTP@PLGA could mainly induce apoptosis of 4T1 cells under US irradiation (1 W/cm^2^ for 3 min), in which the proportion of late apoptosis is higher than that of the early apoptosis. Interestingly, the ratio of early and late apoptosis of 4T1 cells in PTP@PLGA + US treated group is almost the same with that in PP@PLGA + US treated group. As expected, the total proportion of apoptosis that PTP@PLGA caused is significantly higher than that of PP@PLGA under US irradiation, which is consistent with the above CCK-8 and CLSM results. Thus, the US-mediated synergistic therapeutic effect of PTP@PLGA has been proved as a viable way to kill cancer cells *via* inducing apoptosis.

### In vitro ultrasound effect on cancer stem-like cells

As a small portion of cancer cells, the cancer stem-like cells (CSCs) are believed to exhibit higher chemoresistance. Thus, in order to further evaluate the ultrasound effect on CSCs, PP@PLGA was chosen to co-incubated with CSCs under ultrasound stimulation, which were enriched from 4T1 cells in a serum-free culture according to the reported method [19]. The optical microscope images of the origin adherent 4T1 cells and the obtained CSCs, also identified as mammospheres, were shown in Fig. 4F. Then, the obtained mammospheres (CSCs) were treated with PP@PLGA, US and PP@PLGA + US, respectively. As shown in Fig. 4G, the expression of stemness-associated genes (i.e., SOX2, NANOG, OCT4) of CSCs, measured *via* the PCR technique, was significantly higher than that of 4T1 cells as expected, indicating the successful in vitro culture of CSCs. Notably, the expression of the three above genes of CSCs decreased both in US and PP@PLGA + US treated groups.

Furthermore, acetaldehyde dehydrogenase (ALDH), a specific intracellular marker of CSCs, was also assayed using a commercial kit based on flow cytometry, which exhibited a similar trend with the above PCR results. Mechanistically, ALDH can catalyze the probe to generate green fluorescence signal within cells, which is counted by flow cytometry. Diethylaminobenzaldehyde (DEAB) is a specific inhibitor of ALDH for negative control [47]. Specifically, according to Fig. 4H and I, the percentage of ALDH + subpopulation in CSCs was about 9.9%, which was higher than the 4T1 cells. After US and PTP@PLGA + US treatment, the percentage of ALDH + cells decreased to 3.1% and 1.2% respectively. The above results both indicate that the ultrasound effect (sonomechanical and sonodynamic effect) could somehow downregulate the stem-like features of CSCs (Fig. 4J). The similar phenomenon of ultrasound effect on CSCs was also observed in recent reports [38, 48]. Though the mechanism behind needs further investigation, the ultrasound effect still holds the potential to intervene the CSCs fate in vivo to impair their chemoresistance for chemotherapeutics, e.g., TPZ.

### In vivo bioeffects of PTP@PLGA under ultrasound stimulation

**Fig. 5:**
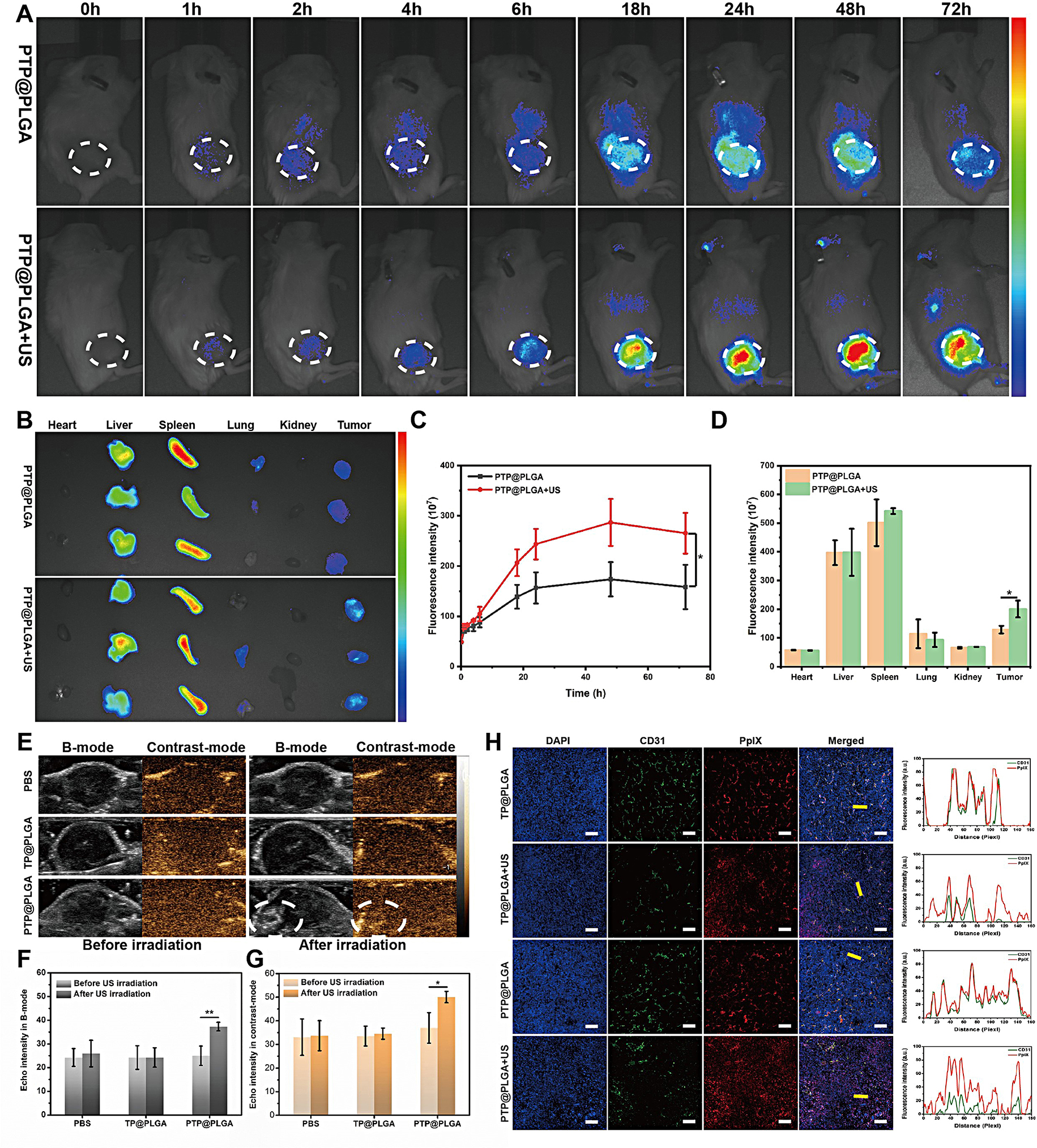
In vivo ultrasound-induced vaporization and the deep penetration of drug. In vivo fluorescence images (**A**) and the quantification of the fluorescence intensity (**C**) in tumor area of 4T1-tumor bearing mice at different time points in PTP@PLGA and PTP@PLGA + US groups. The PTP@PLGA nanodroplets were labeled by DiR dye. Ex vivo fluorescence images (**B**) and the corresponding fluorescence intensity (**D**) of major organs and tumors in PTP@PLGA and PTP@PLGA + US groups. (**E**) In vivo B-mode and CEUS images and (**F**, **G**) the corresponding echo intensity at the tumor sites of tumor-bearing mice intravenously injected with nanodroplets (TP@PLGA and PTP@PLGA, respectively) before and after ultrasound irradiation. US power output: 1 W/cm^2^ for 10 min. (**H**) Fluorescence images and the corresponding intensity line scanning (yellow arrow) profile of tumor tissues of tumor-bearing mice receiving different treatments. The cell nuclei were stained with DAPI (blue fluorescence) and blood vessels were stained with CD31 (green fluorescence). Scale bar = 80 μm

In vivo ultrasound responsive performance and consequent bioeffects of PTP@PLGA, including ADV, drug penetration, and oxygen consumption, were comprehensively evaluated using 4T1-tumor bearing mice *via i.v.* injection. The biodistribution of PTP@PLGA under ultrasound irradiation in vivo was assessed firstly. As shown in Fig. 5A and C, the fluorescence signal was gradually clustered at tumor region and peaked at 48 h in PTP@PLGA + US group and weakened at 72 h. The similar trend was observed in PTP@PLGA group while its fluorescence signal intensity was significantly lower than that in PTP@PLGA + US group. This might attribute to the loosen ECM in tumor after ultrasound irradiation. The ex vivo fluorescence images of major organs and tumor in the two groups were obtained at 72 h post-injection. Consistent with in vivo imaging results, ex vivo biodistribution studies showed that more PTP@PLGA nanodroplets were accumulated at tumor site in PTP@PLGA + US group compared these in PTP@PLGA group (Fig. 5B and D). Then ultrasound imaging (B-mode and contrast-mode) was conducted to verify the intratumor ADV process for PTP@PLGA. As shown in Fig. 5E, without ultrasound irradiation, both TP@PLGA and PTP@PLGA treated groups display no obvious echo signals in the tumor region, which show no difference with the PBS treated group without ultrasound irradiation. In comparison, under ultrasound irradiation (1 W/cm^2^ for 10 min) at the tumor sites, significant echo enhancements in both B-mode and Contrast-mode were observed in the tumor region only for PTP@PLGA treated group, which was further confirmed by the corresponding quantitative results (Fig. 5F and G), indicating that PTP@PLGA could effectively spread into the tumor region *via* blood circulation, then render the intratumor ADV.

Then, the intratumor drug release and penetration were evaluated *via* investigating the distribution of PpIX in the fluorescence images of tumor slices, in which the vascular markers (CD31) were stained in green fluorescence to mark the tumor blood vessels and the tumor cell nuclei were stained in blue fluorescence to mark the tumor cells. As shown in Fig. 5H, without ultrasound irradiation, the red fluorescence of PpIX was highly overlapped with the green fluorescence of CD31 for both TP@PLGA and PTP@PLGA treated groups, indicating that the nanodroplets were mainly restricted around the blood vessels with limited diffusion distance. As expected, only in the PTP@PLGA + US group, enhanced red fluorescence of PpIX was observed, and more importantly the red fluorescence of PpIX was found to be separated from the location of CD31 marked with green fluorescence, indicating a higher release amount and deeper penetration depth of PpIX within tumor tissue.

**Fig. 6:**
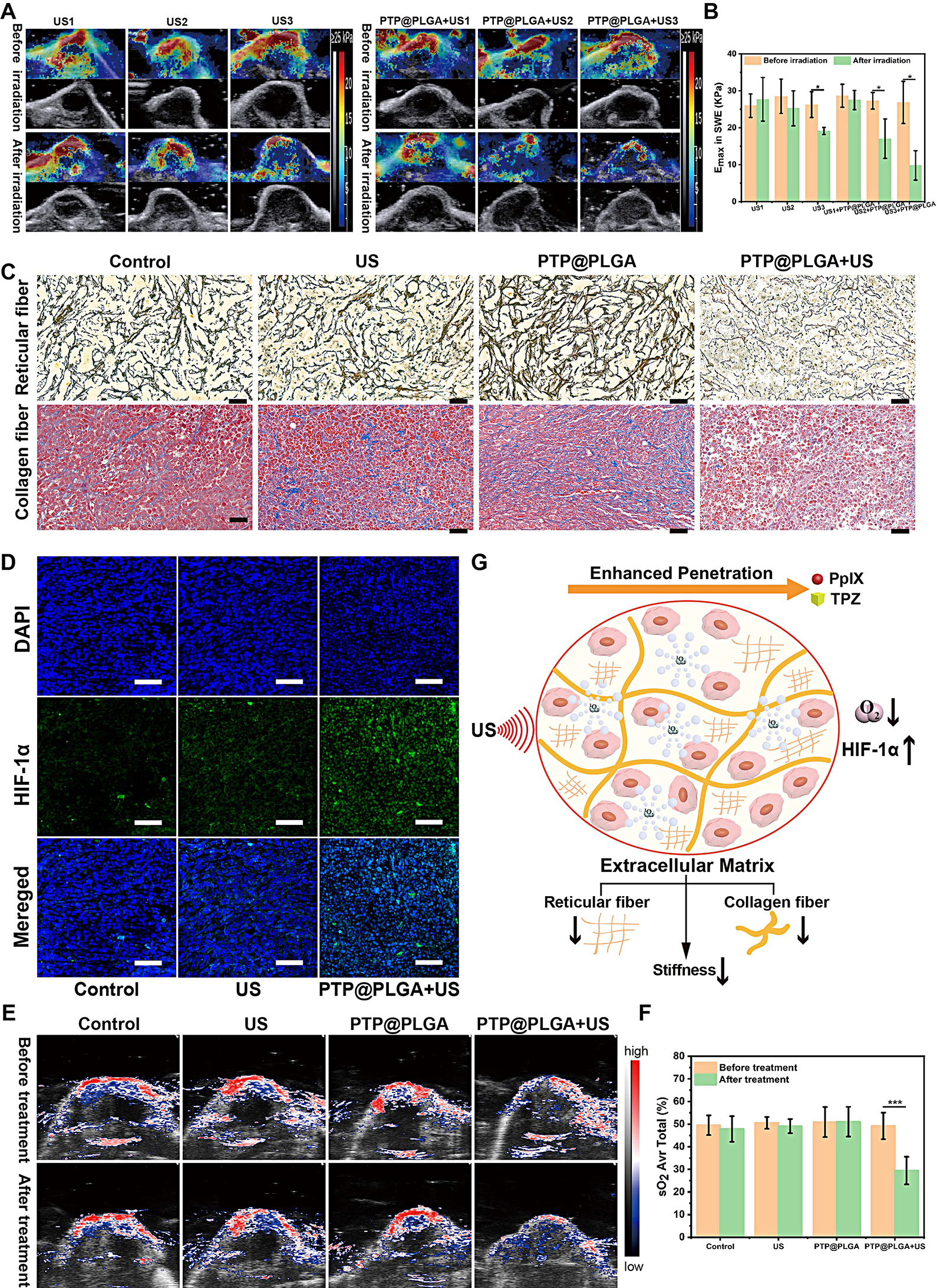
In vivo ultrasound-induced ECM and oxygenation change. (**A**) In vivo B-mode and SWE images of tumors and (**B**) the corresponding maximum Young’s elastic modulus values in different groups before and after different ultrasound irradiation. US1 (0.5 W/cm^2^, 10 min), US2 (1.0 W/cm^2^, 10 min), US3 (1.5 W/cm^2^, 10 min). (**C**) Expression levels of reticular fiber and collagen fiber of tumors in different groups detected by gordon staining and masson staining. Scale bar = 40 μm. (**D**) Ex vivo immunofluorescence staining of HIF-1α of tumor slices from tumor-bearing mice receiving different treatments. US power output: 1 W/cm^2^ for 10 min. Scale bar = 60 μm. (**E**) PA/US imaging of sO_2_ in tumor-bearing mice with different treatments. (**F**) Corresponding average total tumor sO_2_ in mice with different treatments. (**G**) Schematic illustration of the ultrasound effect on extracellular matrix and oxygen level of tumor for enhanced penetration and activation of TPZ

Since the dense ECM forms the main barrier for the deep penetration of chemotherapeutics or nanoparticles, the in vivo ECM changes after different treatments were further evaluated [49]. Firstly, the Young’s elastic modulus of tumor tissue was measured by ultrasound shear-wave elastography (SWE), which is considered to be highly related to the ECM change [50, 51]. The tumor-bearing mice were divided into six group with different treatment under different ultrasound intnesity. As shown in Fig. 6A and B, no significant change of Young’s elastic modulus was observed after treatment with US1, US2 and PTP@PLGA + US1. While, in US3, PTP@PLGA + US2 and PTP@PLGA + US3 treated group, the Young’s elastic modulus decreased after US irradiation. It proved that PTP@PLGA could promote the mechanical effect of ultrasound. Furthermore, as shown in the pathological staining results (Fig. 6C), reticular and collagen fibers were almost intact in US and PTP@PLGA treated group. Whereas, the structure of fibers was incomplete in the PTP@PLGA + US group. Thus, the ADV of PTP@PLGA is believed to destruct the ECM to promote the drug penetration into the hypoxic regions of tumor due to the sonomechanical effect [52, 53].

In addition to its violent sonomechanical effects, sonodynamic effect induced oxygen consumption in tumor was further evaluated for PTP@PLGA *via* measuring the expressed level of HIF-1α, which is positively correlated to the intratumor hypoxic level [54]. As shown in Fig. 6D, ex vivo immunofluorescence staining of HIF-1α indicates a significant up-regulation of HIF-1α in PTP@PLGA + US treated group comparing to that of PBS and US treated group, validating that with the PpIX component, PTP@PLGA could enable the in vivo sonodynamic effect to consume the intratumor oxygen, resulting in elevated hypoxia status to activate TPZ. The change of oxygen content induced by sonodynamic effect was further measured directly by photoacoustic imaging in a Vevo LAZER system. The tumor-bearing mice were divided into four groups: Control, US, PTP@PLGA, PTP@PLGA + US. As shown in Fig. 6E and F, the sO_2_ decreased only in the group treated with PTP@PLGA + US, which decreased from 49.1% to about 29.5% after ultrasound 5 min post PTP@PLGA administration. There was no significant difference of oxygen content in another three groups before and after ultrasound irradiation. It proved that PTP@PLGA could enable the in vivo sonodynamic effect to consume the intratumor oxygen and resulted in elevated hypoxia status.

To sum up, as shown in Fig. 6G, the PTP@PLGA can induce remarkable in vivo bioeffects under US stimulation *via* the sonomechanical and sonodynamic effect, including extracellular matrix destruction and oxygen consumption, which result in enhanced penetration and activation of TPZ.

### In vivo evaluation of antitumor efficacy

**Fig. 7:**
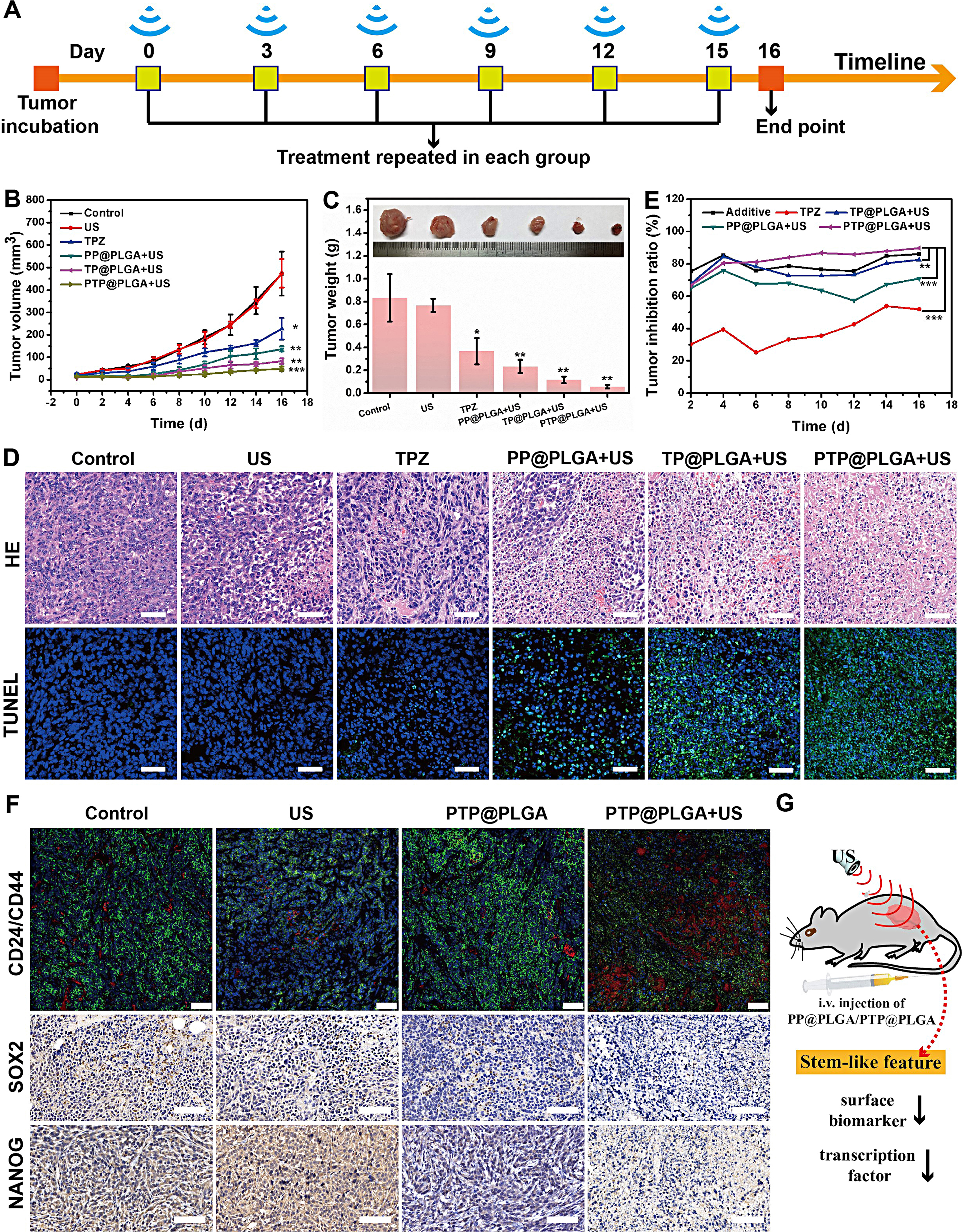
In vivo synergetic therapy and stem-like feature change. (**A**) Schematic illustration of the in vivo treatment procedure on 4T1-tumor bearing mice. (**B**) Tumor growth profiles of 4T1-tumor bearing mice in different treating groups. (**C**) Average weights of tumors collected from different groups 16 days after treatments and their representative images. (**D**) H&E and TUNEL staining of tumor tissues from different groups. Scale bar = 50 μm. (**E**) The calculated tumor inhibition ratios of different treating groups and the calculated additive tumor inhibition ratio of group 3) and 4). (**F**) Immunofluorescence staining of CD24 (red)/CD44(green) in 4T1 tumor-bearing mice receiving different treatments. Scale bar = 50 μm. Immunohistochemistry staining of the SOX2, and NANOG in 4T1 tumor-bearing mice receiving different treatments. Scale bar = 80 μm. (**G**) Schematic illustration of the ultrasound effect on downregulating the stemness feature of tumor tissue

To evaluate the in vivo antitumor performance of PTP@PLGA, 4T1-tumor bearing mice were divided into six groups for different treatments. For group 4, 5 and 6, as illustrated in Fig. 7A, ultrasound irradiation was applied at the tumor sites 5 min post *i.v*. injection of different nanodroplets, which were repeated every three days. As shown in Fig. 7B and C and Figure S10, the tumors of mice in group 2 grew continuously like group 1. Furthermore, inhibition effects of tumor growth the TP@PLGA, PP@PLGA and PTP@PLGA groups were also evaluated, which displayed no significant difference compared with control groups (Figure S11A and S11B). Comparatively, as expected, group 3, 4, 5 and 6 all exhibited inhibition effect of tumor growth and the most significant inhibition effect was found in group 6, indicating that the combined therapeutic effect of TPZ and sonodynamic effect was superior to that of TPZ or sonodynamic effect alone. Consistent with the above tumor inhibition results, the most severe tumor cell damage was observed in excised tumors of group 6 as shown in the H&E and TUNEL staining images (Fig. 7D).

Then, the tumor inhibition ratios (f) for group 3, 4, 5 and 6 were calculated by the following equation:

f_i_=V_i_/V_1_ × 100%, i = 3,4,5,6.

V is the mean tumor volume in each group. And the additive inhibition ratio (fa) of group 3 and 4) was calculated by the following equation:

f_a_= (1-f_3_×f_4_ )×100%.

As shown in Fig. 7E, the tumor inhibition ratio of group 6 was obviously higher than that of group 3, 4, 5 and their additive one, further confirming the synergistic effect of TPZ and ultrasound effect in group 6.

Furthermore, the ultrasound effect on the stem-like feature of cancer cells was also evaluated in vivo. The tumor-bearing mice were randomly divided into four groups (*n* = 3 per group) for different treatments: (1) saline; (2) US; (3) PTP@PLGA; (4) PTP@PLGA + US. As shown in Fig. 7F, the stemness-related feature expression of 4T1 cells, including surface biomarker (CD24 and CD44) and transcription factor (NANOG and SOX2) were evaluated. The CD24/CD44 staining were visible in the cytomembrane and the SOX2/NANOG-specific staining were in the nucleus of cancer cells. The expression of these CD44, SOX2 and NANOG markers in control, US and PTP@PLGA groups were similar and high. Notably, the PP@PLGA + US treated group showed a decreased expression in the cytomembrane of CD44 and the nucleus of SOX2/NANOG. On the contrary, the expression of surface marker CD24 was increased only in the PTP@PLGA + US treated group. The promoted suppression of CSC stemness might be attributed to the combined sonomechanical and sonodynamic effects of PP@PLGA and PP@PLGA, which might also reverse the chemoresistance of 4T1 cells to further potentiate TPZ.

At last, the biosafety of different treatments was evaluated by changes of body weight, hematology biochemistry tests and histological section. The body weights in all groups changed slightly within the 16-day observation period (Figure S12). And the results of blood biochemical examination indicated that all the treatments caused no significant side effects and no hepatic or kidney dysfunction was found after the treatments (Figure S13). Moreover, no evident abnormalities were observed in pathological HE slices of the organs including heat, liver, spleen, lung and kidney as compared with control group (Figure S14). All these results confirmed high biocompatibility of PTP@PLGA nanodroplets and their great potential for in vivo therapy.

## Conclusion

In summary, an intratumor potentiating strategy for hypoxia-activated prodrug, i.e., tirapazamine (TPZ), is proposed based on ultrasound-responsive nanodroplets (PTP@PLGA) *via* the sonodynamic and sonomechanical effect, which are constructed *via* a multi-step emulsion method. With the perfluoropropane core, PTP@PLGA can undergo explosion under ultrasound irradiation due to the acoustic droplet vaporization as visualized by the ultrasound imaging, releasing the loaded TPZ and PpIX. Meanwhile, on one hand, the sonomechanical effect (cavitation) is largely enhanced, concurrently reducing the interstitial fluid pressure and loosening the extracellular matrix of tumor for the deep penetration of TPZ into the highly hypoxic region. On the other hand, the intratumor oxygen is consumed to generate the cytotoxic singlet oxygen *via* the sonodynamic effect of PpIX, resulting in the full activation of TPZ especially in the normoxic region near blood vessels. Notably, the stemness-related factor expression of cancer cell is also downregulated due to the combined ultrasound effect both in vitro and in vivo. Thus, the TPZ is expected to be fully effective in tumor, which, together with the singlet oxygen, can successfully retard the tumor growth. This work offers a general strategy to strengthen the intratumor efficacy of HAP *via* the ultrasound-based nanotechnology with promising prospect for clinical research.

## Materials and methods

### Materials

PEGylatd poly (lactic-co-glycolic acid) (PLGA, lactide: glycolide = 50: 50, 20,000 Da, PEG, 5000 Da) were purchased from Shan-dong Key Laboratory of Medical Polymer Materials (Shandong, China). Protoporphyrin IX (PpIX) and tirapazamine (TPZ) were obtained from MedChemExpress (Shanghai, China). Perfluoropentane, 1,3-Diphenylisobenzofuran (DPBF), 2’,7’-Dichlorodihydrofluorescein diacetate (DCFH-DA) and propidium iodide (PI) were obtained from Sigma Aldrich (St. Louis, USA). Hoechst 33,342 was gained from Beyotime Biotechnology (China). Calcein-AM (CAM) was purchased from Santa Cruz Biotechnology (TX, USA). Rabbit polyclonoal HIF-1α antibody were purchased from Novus Biologicals (Littleton, USA) and Anti-CD31 antibody were obtained from abcam (Cambridge, UK). Anti-Nanog, Anti-Oct4 and Anti-SOX2 antibodies were purchased from abcam (Cambridge, UK). Anti-CD24 and Anti-CD44 were gained from ABclonal Technology (Wuhan, China). ALDEFLUOR kit was gained from STEMCELL Technologies (Vancouver, Canada). Hypoxia/Oxidative stress detection kit was purchased from Enzo Life Sciences (NY, USA).

### Preparation of PTP@PLGA nanodroplets

The PFP-TPZ-PpIX@PLGA-PVA (PTP@PLGA) nanodroplets were prepared *via* a three-step emulsion method [40]. Firstly, 0.2 mL of PFP was emulsified with 0.2 mL of TPZ aqueous solution (10 mg/mL) in ice bath by a sonicator (Heat System Inc) at 150 W for 1 min. Secondly, 10 mg of PLGA and 1 mg of PpIX were added into 2 mL of chloroform, which were then transferred to the above PFP-TPZ mixture and sonicated at 150 W for 3 min. Thirdly, 10 mL of PVA solution (4% w/v) was added and sonicated at 100 W for another 3 min. Finally, the emulsified solution was stirred magnetically for 6 h to extract chloroform completely and centrifuged for further use. The PFP-PpIX@PLGA-PVA (PP@PLGA) nanodroplets, TPZ-PpIX@PLGA-PVA (TP@PLGA) nanodroplets were obtained *via* the same procedure without the addition of TPZ or PFP.

### Characterization of PTP@PLGA nanodroplets

The structure and morphology of PTP@PLGA nanodroplets were characterized by transmission electron microscope (TEM, JEM-2100 F) and scanning electron microscope (SEM). The distribution of C, N, O and F was analyzed by TEM (JEM-F200). The size distribution and ζ-potential were measured by Malvern instrument (Nano ZS90). The UV-vis spectra and fluorescence spectra were recorded by Shimadzu UV-2600 spectroscopy and Shimadzu RF-6000 spectroscopy respectively. And the loading efficiency and loading content of TPZ were calculated by the following equation: Loading efficiency (%) = (total TPZ-unbound TPZ)/total TPZ; Loading content (%) = (total TPZ-unbound TPZ)/total nanodroplets. The same calculational formula was applied to PpIX as well. And the photo-stability of PTP@PLGA nanodroplets and free PpIX were assayed through recording the change of UV-vis and fluorescence spectra for 7 days in dark and light respectively.

### In vitro ultrasound imaging and ultrasound-induced TPZ release

To confirm the successful encapsulation of PFP, the morphological change of PTP@PLGA was first visualized by optical microscope (Olympus, Tokyo) at different temperatures. Then, US imaging was applied to observe TP@PLGA and PTP@PLGA under ultrasound irradiation (1 W/cm^2^ for 30 s). B-mode and contrast-mode ultrasound (CEUS) images were captured by ultrasonic instrument (Resona 7, China) and the echo intensities were measured by an US image analyzer (DFY software, Institute of Ultrasound Imaging of Chong Qing Medical University). In vitro TPZ release from TP@PLGA and PTP@PLGA were measured by dialysis method. 1 mL of nanodroplets dispersion were packaged into dialysis bags and incubated in 50 mL PBS with or without ultrasound irradiation (1 W/cm^2^ for 5 min, every 50 min). At different time intervals, dialysate was collected for TPZ detection and replaced with the same volume of fresh PBS. The TPZ concentration was measured by UV-vis spectrometer.

### In vitro sonodynamic effect of PTP@PLGA

^1^O_2_ and ^.^OH generation were evaluated by TEMP and DMPO as trapping reagents. For ^1^O_2_ detection, 5µL TEMP was mixed with 50µL PTP@PLGA (the concentration of PpIX was 20 µg/mL) and irradiated by US (1.5 W/cm^2^ for 60 s). As for ^.^OH generation, all the procedures were the same as ^1^O_2_ detection except the reagent was replaced with DMPO. The signals of ^1^O_2_ and ^.^OH were measured by the ESR spectrometer. DPBF was further used to evaluate the in vitro singlet oxygen generation of PTP@PLGA under ultrasound irradiation [55]. Briefly, DPBF in DMF (10 µL, 10 mM) was added to 2 mL of PP@PLGA, TP@PLGA and PTP@PLGA with the same concentration of PpIX (20 µg/mL), respectively. Then, the solution was exposed to ultrasound with different power intensities (0.5, 1 and 1.5 W/cm^2^ for 60 s) or different durations (1 W/cm^2^ for 30, 60 and 120 s), respectively. The absorption intensity of DPBF at 410 nm was measured by UV-vis spectrometer. Meanwhile, the oxygen level before and after ultrasound irradiation was also measured using a portable dissolved oxygen meter (JPB-607 A). The PP@PLGA, TP@PLGA and PTP@PLGA nanodroplets (PpIX, 20 µg/mL) were dissolved in degassed water and then exposed to ultrasound with different power intensities (60 s, 0.5, 1 and 1.5 W/cm^2^) or different durations (1 W/cm^2^, 30, 60, 120 and 180 s), respectively. And DCFH-DA was employed to evaluate the intracellular singlet oxygen generation. 4T1 cells were co-incubated with PBS, PP@PLGA, TP@PLGA and PTP@PLGA with the same concentration of PpIX (20 µg/mL) for 4 h, respectively. Then, the above 4T1 cells were incubated with DCFH-DA for 0.5 h, which were subsequently irradiated by ultrasound (1 W/cm^2^ for 180 s). After incubation for another 0.5 h, these cells were collected for analysis *via* flow cytometry (CytomicsTM FC500 cytometer, Beckman Coulter) and confocal laser scanning microscope (FV 1000, Olympus). The intracellular ROS and hypoxia generation were further assessed by using hypoxia/Oxidative stress detection kit. The 4T1 cells divided into four groups: control, US, PTP@PLGA and PTP@PLGA + US. These cells were treated with PBS or PTP@PLGA for 4 h and subsequently irradiated with or without ultrasound. Then the cells in different treatment groups were imaged by CLSM.

**Cell culture and animal model** 4T1 cells were cultured in PRMI 1640 medium with 10% fetal bovine serum and 1% penicillin/streptomycin at 37 ℃ in atmosphere with 5% CO_2_. For the formation of mammospheres, 4T1 cells were seeded into ultra-low attachment six-well plates (Corning, USA) and incubated with DMEM/F12 medium supplemented with B-27, 20 ng/mL of fibroblast growth factors-basic, 20 ng/mL epidermal growth factor, and 5 µg/mL insulin for about 10 days [14, 19].

All animal studies were conducted under a protocol approved by the Animal Ethics Committee of Fudan University (2019 JS-144). And the 4T1 breast cancer xenografts were formed by subcutaneous administration of 1 × 106 4T1 cells in 100 µL of serum-free medium into the back of the female BALB/c mice. And all experiments were carried out when the tumor volumes reached about 80 mm^3^.

### Synergetic therapy in cell level

To evaluate the cytotoxicity of TP@PLGA, PP@PLGA and PTP@PLGA nanodroplets, 4T1 cells were incubated with them at different concentrations (the concentration of PpIX is 7.5, 15 and 30 µg/mL) for 24 h. And cell viabilities were determined by CCK-8 assay. To test the therapeutic effects, 4T1 cells were treated with different concentrations of TP@PLGA, PP@PLGA and PTP@PLGA nanodroplets for 4 h, which were then exposed to ultrasound irradiation (1–2 W/cm^2^) for 3 min. After another 24 h incubation, the cell viabilities were determined by CCK-8 kit, flow cytometry and further assessed by confocal fluorescence microscopy after co-staining with CAM (2 µM) and PI (4 µM) [54].

### In vitro stem-like feature evaluation

The stem-like feature of cancer cells, including the transcription factor (SOX2, NANOG and OCT4) and acetaldehyde dehydrogenase (ALDH), was evaluated for adherent 4T1 cells and mammospheres (cancer stem-like cells) under different treatments (PP@PLGA, US and PP@PLGA + US, PpIX, 20 µg/mL), US power output: 1 W/ cm^2^ for 3 min), which were determined using the real-time polymerase chain reaction (PCR) system (Thermo Fisher, America) and the ALDEFLUOR kit *via* flow cytometry, respectively. The primer sequences of the targeted genes (SOX2, NANOG and OCT4) were shown in Table S1.

### In vivo ultrasound imaging and analysis of drug distribution

For in vivo biodistribution of PTP@PLGA, the tumor-bearing mice were randomly divided to two groups: PTP@PLGA and PTP@PLGA + US. The PTP@PLGA were intravenously injected with the same content of PLGA (2 mg/mL) and the ultrasound irradiation (1 W/cm^2^ for 10 min) was applied at the tumor sites 5 min post injection. The fluorescence images were obtained at different time points (pre, 1, 2, 4, 6, 18, 24, 48 and 72 h) by fluorescence imaging system. At 72 h post injection, the tumor tissues and main organs were collected to evaluate the fluorescence intensities.

For in vivo US imaging, the tumor-bearing mice were randomly divided to three groups and intravenously injected of PBS, TP@PLGA and PTP@PLGA with the same content of PLGA (2 mg/mL), respectively. Then, ultrasound irradiation (1 W/cm^2^ for 10 min) was applied at the tumor sites 5 min post intravenously injecting. Meanwhile, the B-mode and contrast-mode US (CEUS) images of tumors were captured by ultrasonic instrument (Resona 7, China) and the echo intensities were measured by an US image analyzer (DFY software, Institute of Ultrasound Imaging of Chong Qing Medical University). The in vivo drug release from nanodroplets were observed and analyzed by detecting the fluorescence of PpIX as the fluorescence of TPZ was too weak to be detected. The tumor-bearing mice were randomly divided into four groups for different treatments: (1) TP@PLGA; (2) TP@PLGA + US; (3) PTP@PLGA; (4) PTP@PLGA + US. For group 2 and 4, ultrasound irradiation (1 W/cm^2^ for 10 min) was applied at the tumor sites of the mice 5 min post *i.v.* injection of the nanodroplets (PpIX: 9 mg/kg; TPZ: 6 mg/kg). Then, after 24 h, tumors from all the groups were harvested and stained with anti-CD31 antibody and DAPI to label blood vessel and cell nucleus, respectively. And the PpIX distribution was observed by confocal laser scanning microscope.

### In vivo oxygen level evaluation in tumor

To assay the sonodynamic effect on hypoxia status in tumors, HIF-1α antibody was used to stain ex vivo tissue for immunostaining analysis. The tumor-bearing mice were randomly divided into three groups (*n* = 3 per group) for different treatments: (1) saline; (2) US; (3) PTP@PLGA + US. For mice in group 3, US irradiation (1 W/cm^2^ for 10 min) was applied at the tumor sites of mice 5 min post intravenous (*i.v.*) injection of nanodroplets (PpIX: 9 mg/kg; TPZ: 6 mg/kg). 24 h later, all the tumors were collected for HIF-1α analysis. The hypoxia status was further evaluated by photoacoustic imaging (Vevo LAZER) by measuring the change of blood oxygen saturation (sO_2_) at tumor region. The tumor-bearing mice were divided into four groups: (1) Control; (2) US; (3) PTP@PLGA; (4) PTP@PLGA + US. Ultrasound (1.0 W/cm^2^, 10 min) was applied at the tumor sites of mice 5 min post intravenous (*i.v.*) injection of PTP@PLGA nanodroplets (PpIX: 9 mg/kg; TPZ: 6 mg/kg). The photoacoustic images of tumor site and quantitative analysis of signal intensities were performed before and after ultrasound irradiation.

### In vivo assessment of tumor tissue

The tumor-bearing mice were randomly divided into six groups (*n* = 3 per group) for different treatments: (1) US1(0.5 W/cm^2^, 10 min); (2) US2 (1.0 W/cm^2^, 10 min); (3) US3 (1.5 W/cm^2^, 10 min); (4) PTP@PLGA + US1; (5) PTP@PLGA + US2; (6) PTP@PLGA + US3. Ultrasound was applied at the tumor sites of mice 5 min post intravenous (*i.v.*) injection of PTP@PLGA nanodroplets (PpIX: 9 mg/kg; TPZ: 6 mg/kg). Ultrasound shear-wave elastography (SWE) was used to quantitatively evaluate the Young’s elastic modulus of tumor tissue for mice before and after US irradiation. At the third day post treatments, the tumors in each group were collected and evaluated by Gordon-Sweets staining and Masson staining to indicate the changes of ECM structure including reticular fiber and collagen fiber.

### In vivo synergetic therapy

The tumor-bearing mice were randomly divided into six groups (*n* = 6 in each group) for different treatments: (1) control; (2) US only; (3) TPZ (4) PP@PLGA + US; (5) TP@PLGA + US; (6) PTP@PLGA + US, in which saline, TPZ, PP@PLGA, TP@PLGA and PTP@PLGA were all intravenously injected. For group 4, 5 and 6, US irradiation (1 W/cm^2^ for 10 min) was applied at the tumor sites of mice 5 min post intravenous (*i.v.*) injection of different nanodroplets (PpIX, 9 mg/kg; TPZ, 6 mg/kg). All the treatments for all the mice in each group were repeated every three days and the therapeutic efficacy was evaluated in 16 days by recording the tumor volumes and body weights of mice in each group. Tumor volume was calculated using the equation: tumor volume = length × (width)^2^ / 2. After 16 days, tumors and major organs were collected for hematoxylin and eosin (HE) and transferase-mediated deoxyuridine triphosphatebiotin nick end labeling (TUNEL) staining to evaluate the therapeutic outcome.

### In vivo assessment of stemness-related factor expression

The stem-like feature of cancer cells, including the surface biomarker (CD24 and CD44), transcription factor (NANOG and SOX2) was evaluated by immunofluorescence and immunohistochemical analysis respectively. The tumor-bearing mice were randomly divided into four groups (*n* = 3 per group) for different treatments: (1) saline; (2) US; (3) PTP@PLGA; (4) PTP@PLGA + US. For mice in group 4, US irradiation (1 W/cm^2^ for 10 min) was applied at the tumor sites of mice 5 min post intravenous (*i.v.*) injection of nanodroplets (PpIX: 9 mg/kg; TPZ: 6 mg/kg). After 3 days, tumors were collected for abovementioned immunohistochemical staining.

### Statistical analysis

All data were expressed as mean ± standard deviation. Statistical comparisons between two groups were conducted by Student’s t test. And *p < 0.05, **p < 0.01, ***p < 0.005 and ****p < 0.001 were considered significant.

### Electronic supplementary material

Below is the link to the electronic supplementary material.

Supplementary Material 1

## Data Availability

No datasets were generated or analysed during the current study.
