# Supplementary material for: Sonodynamic and sonomechanical effect on cellular stemness and extracellular physicochemical environment to potentiate chemotherapy
Source: J Nanobiotechnology. 2024 Jun 21;22:358. doi: 10.1186/s12951-024-02623-0 (PMC11191306; doi:10.1186/s12951-024-02623-0)
Supplement: Supplementary file 1 — Supplementary Material 1 [file 12951_2024_2623_MOESM1_ESM.docx]

**Supporting Information**


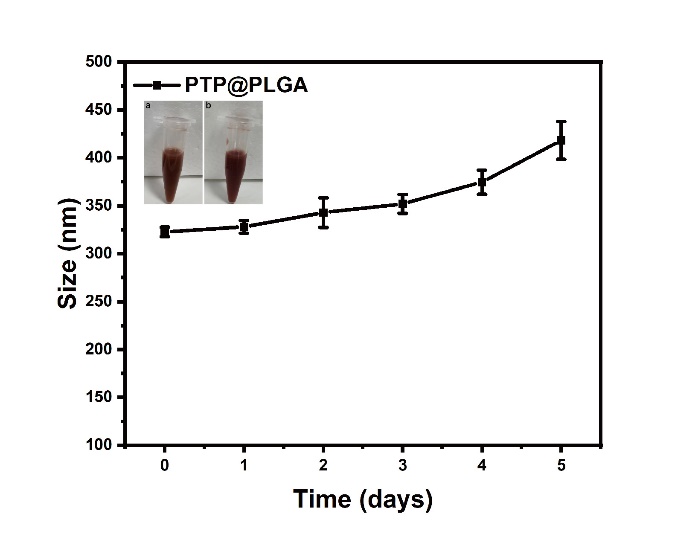


**Figure S1.** The size change of PTP@PLGA during storage in 5 days and photographs of PTP@PLGA (insert) at 0 (a) and 5^th^ (b) day.


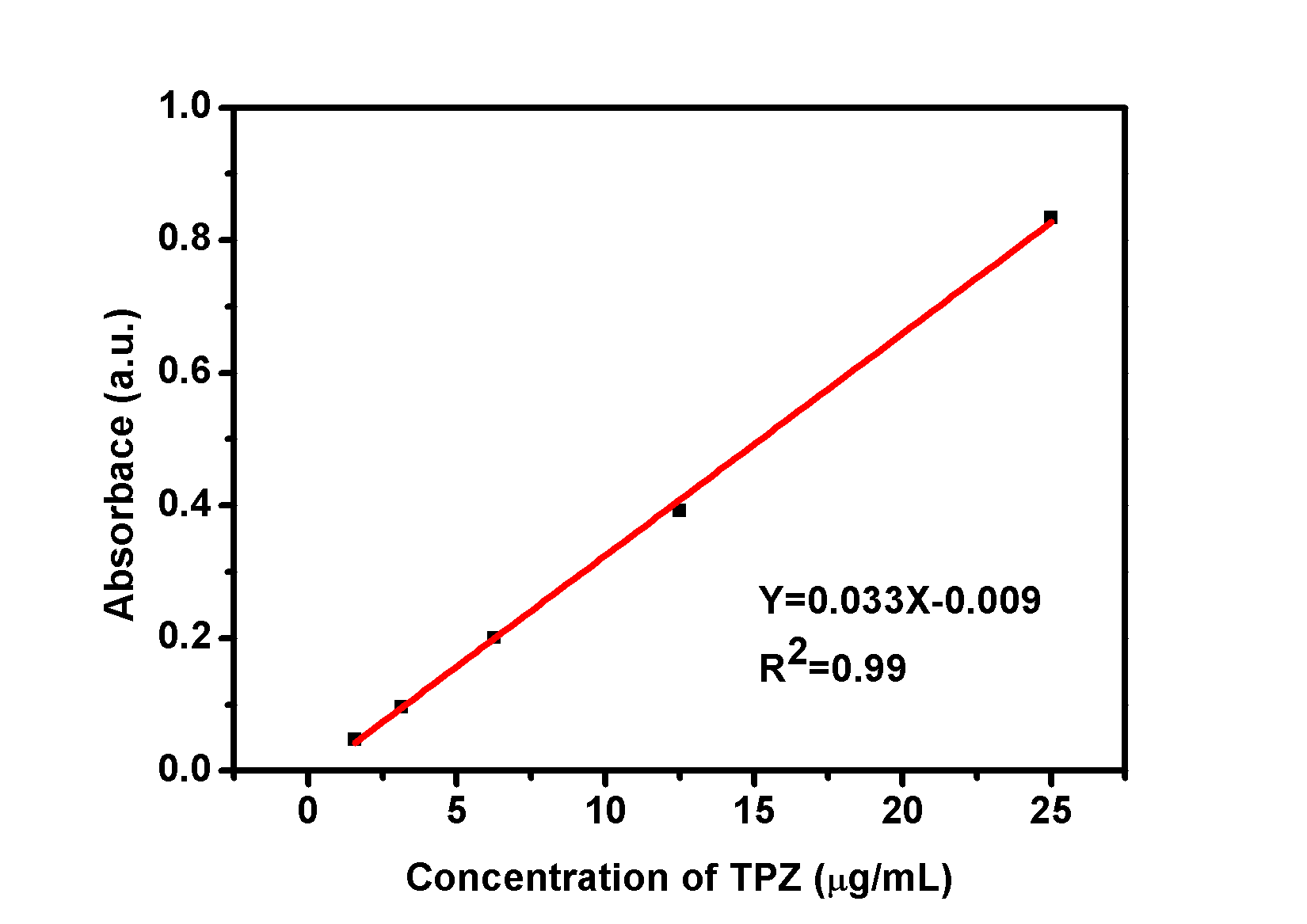


**Figure S2**. The calibration curve of TPZ obtained from the absorbance at 459 nm with different concentration of TPZ.


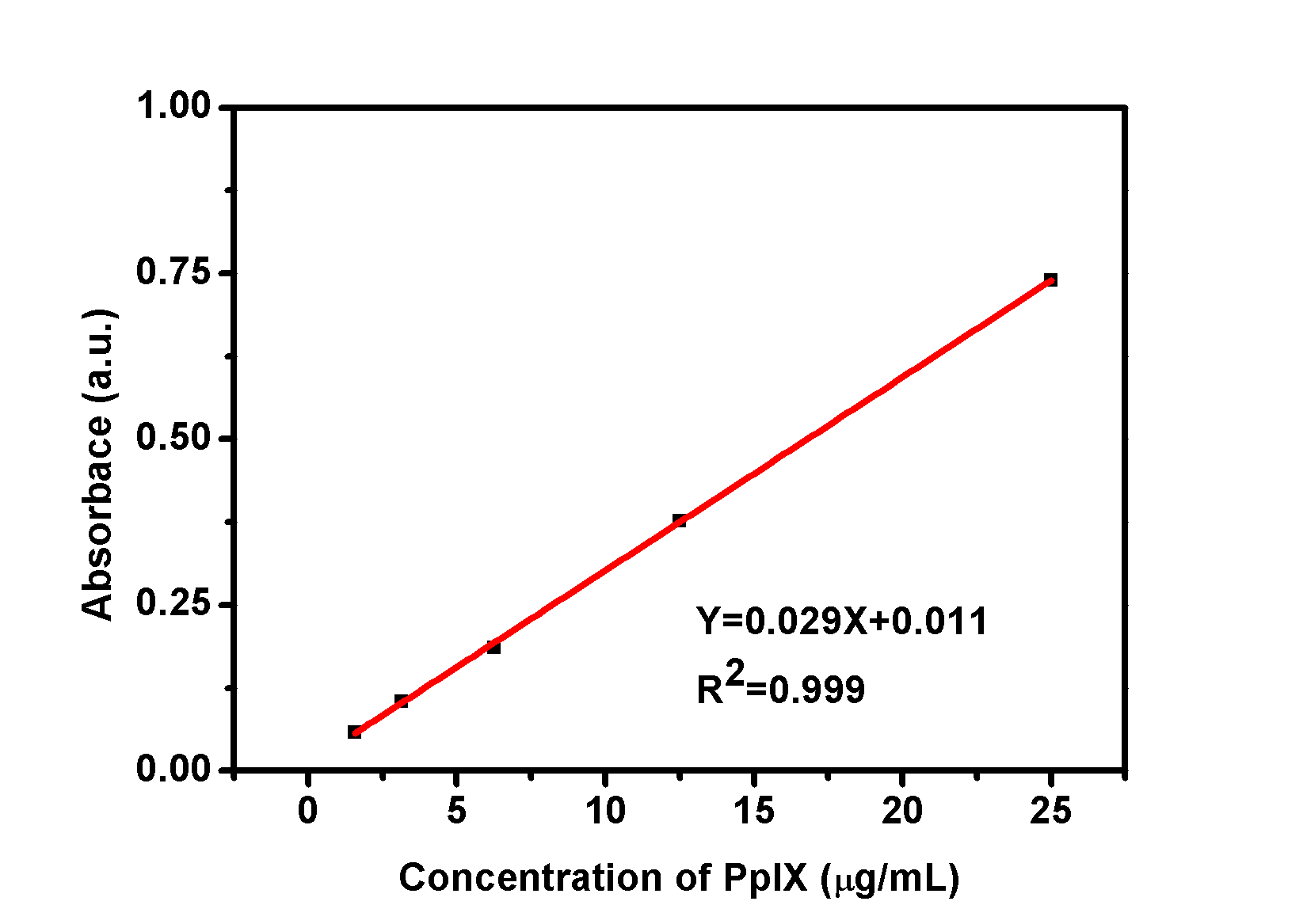


**Figure S3.** The calibration curve of PpIX obtained from the absorbance at 505 nm with different concentration of PpIX.


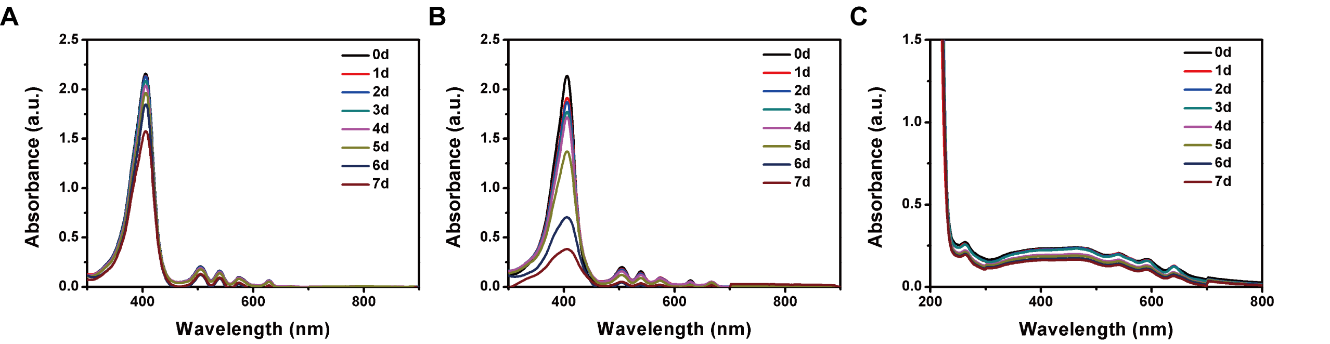


**Figure S4.** (A) UV-Vis spectra of free PpIX in the dark for 7 days. (B) UV-Vis spectra of free PpIX in the light for 7 days. (C) UV-Vis spectra of PTP @PLGA nanodroplets in the light for 7 days.


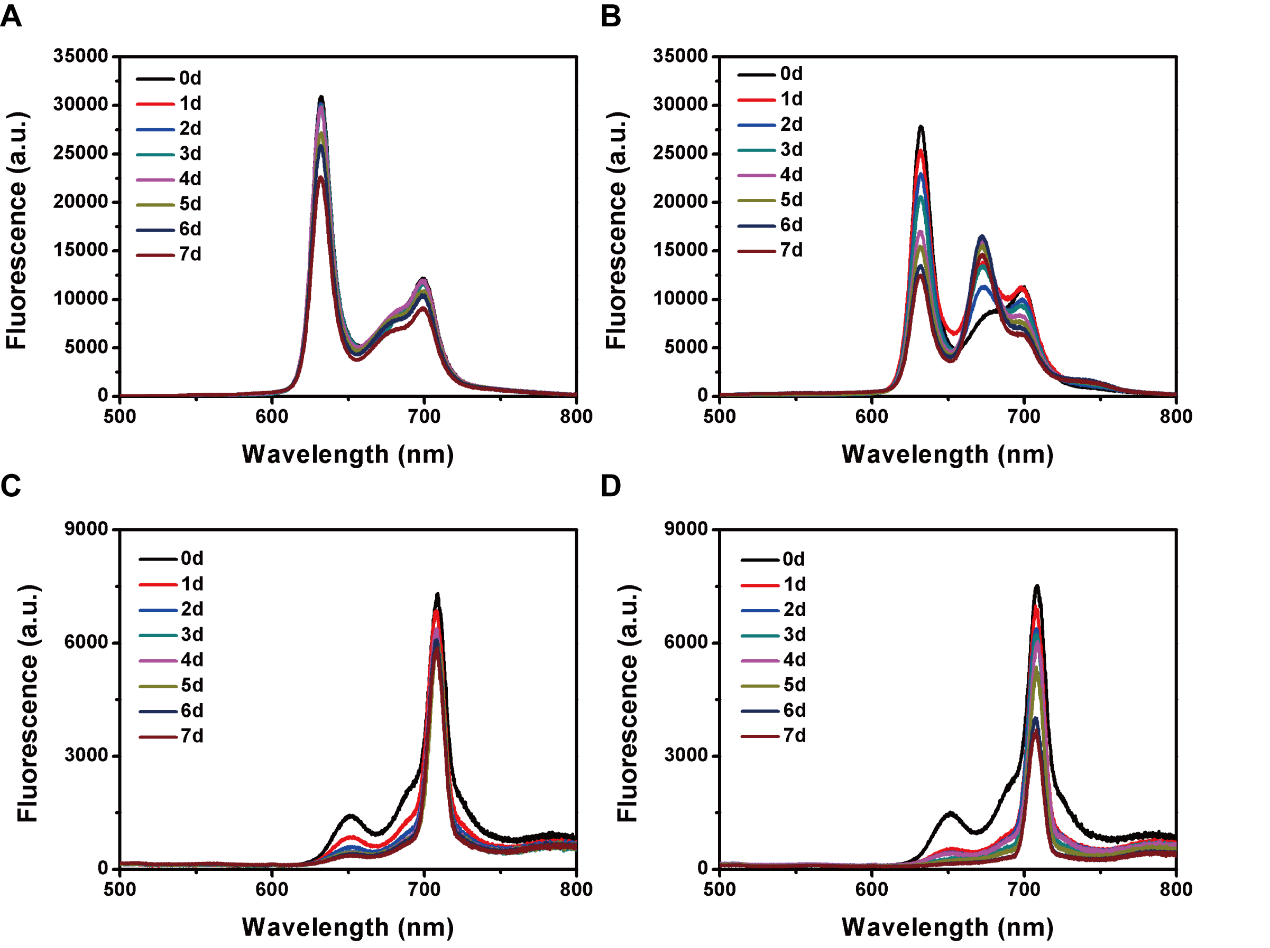


**Figure S5.** (A) Fluorescence spectra of free PpIX in the dark for 7 days. (B) Fluorescence spectra of free PpIX in the light for 7 days. (C) Fluorescence spectra of PTP @PLGA nanodroplets in the dark for 7 days. (D) Fluorescence spectra of PTP @PLGA nanodroplets in the light for 7 days.


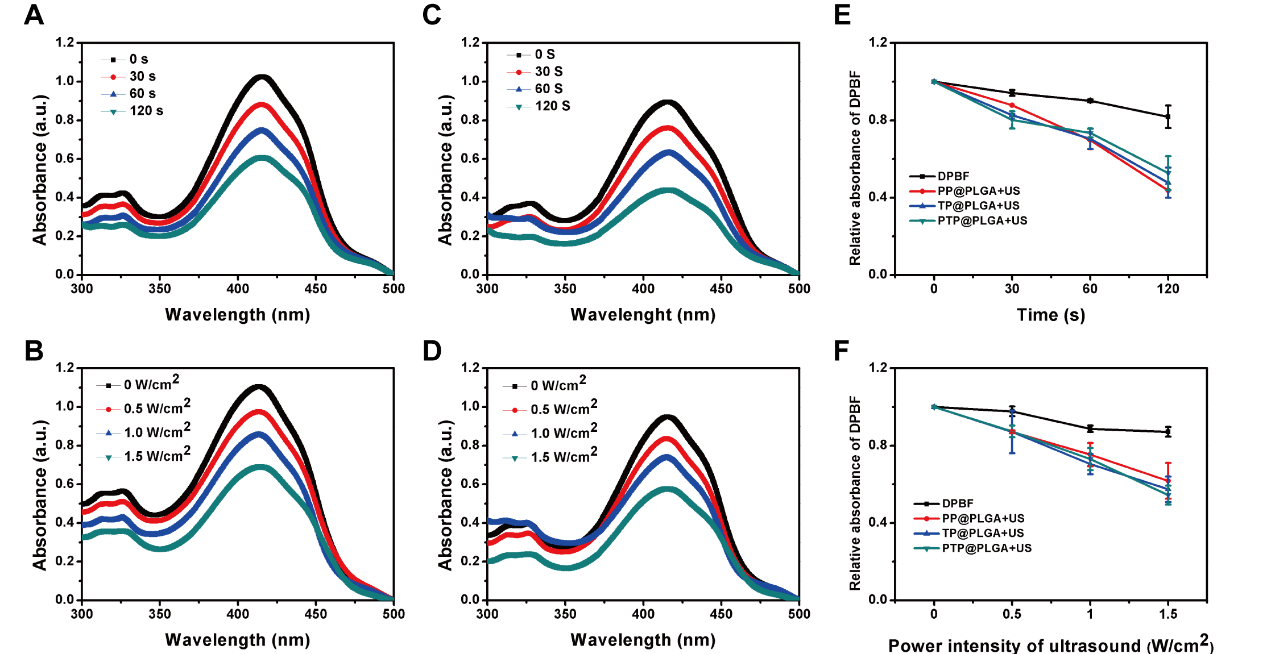


**Figure S6.** (A) The absorption of DPBF in PP@PLGA nanodroplets under ultrasound irradiation with different durations or with (B) different power intensities. (C) The absorption of DPBF in TP@PLGA nanodroplets under ultrasound irradiation with different durations or with (D) different power intensities. (E) Relative absorption of DPBF in different solutions under ultrasound irradiation with different durations. (F) Relative absorption of DPBF in different solutions under ultrasound irradiation with different power intensities.


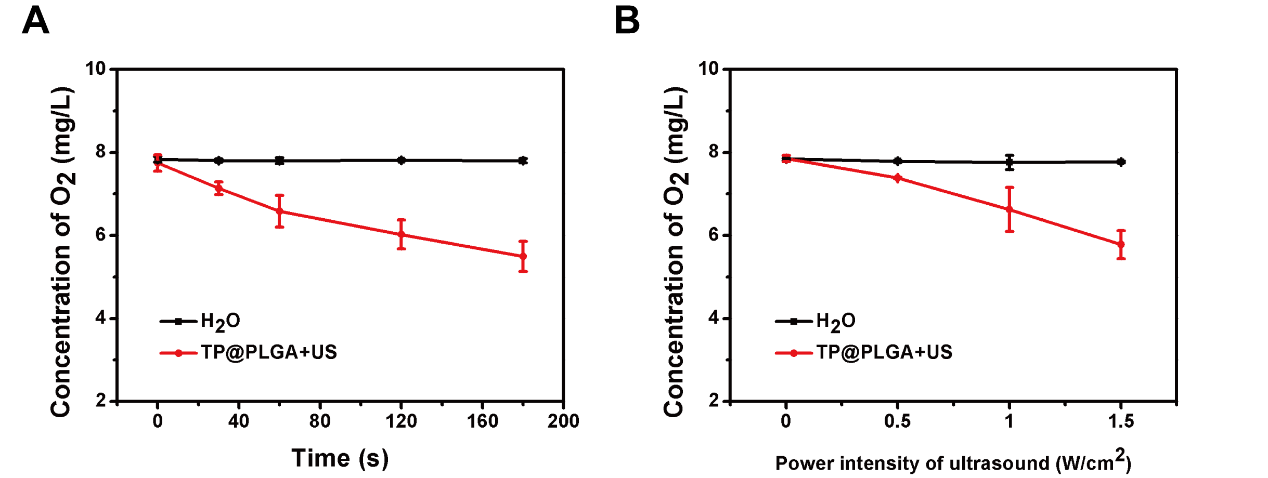


**Figure S7.** (A) The change of oxygen concentration in TP@PLGA nanodroplets under ultrasound irradiation with different durations or with (B) different power intensities.


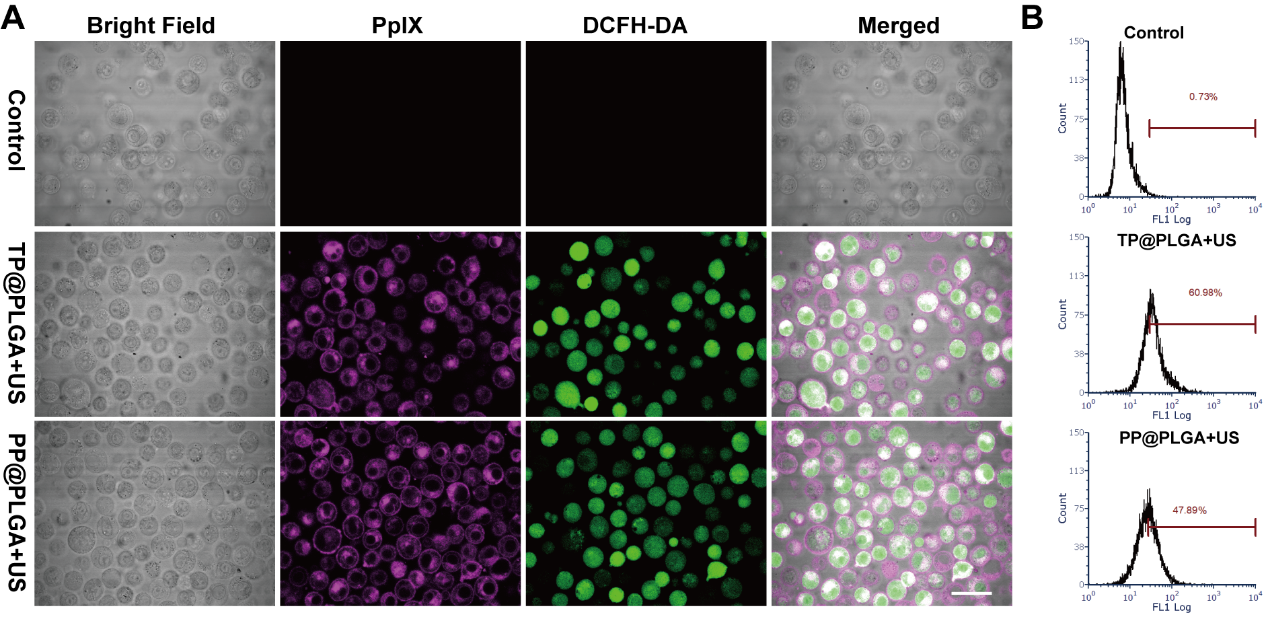


**Figure S8.** (A) Intracellular ROS dection by CLSM in control and TP@PLGA+US and PP@PLGA+US group. Scale bar = 40 μm. (B) Flow cytometry analysis of ROS generation in 4T1 cells in control and TP@PLGA+US and PP@PLGA+US group respectively.

**
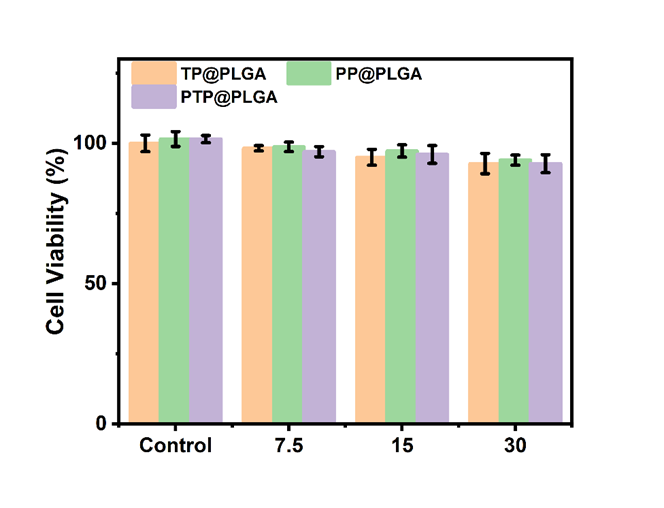
**

**Figure S9.** Cytotoxicity of TP@PLGA, PP@PLGA and PTP@PLGA with different concentration of PpIX to 3T3 fibroblast cells.


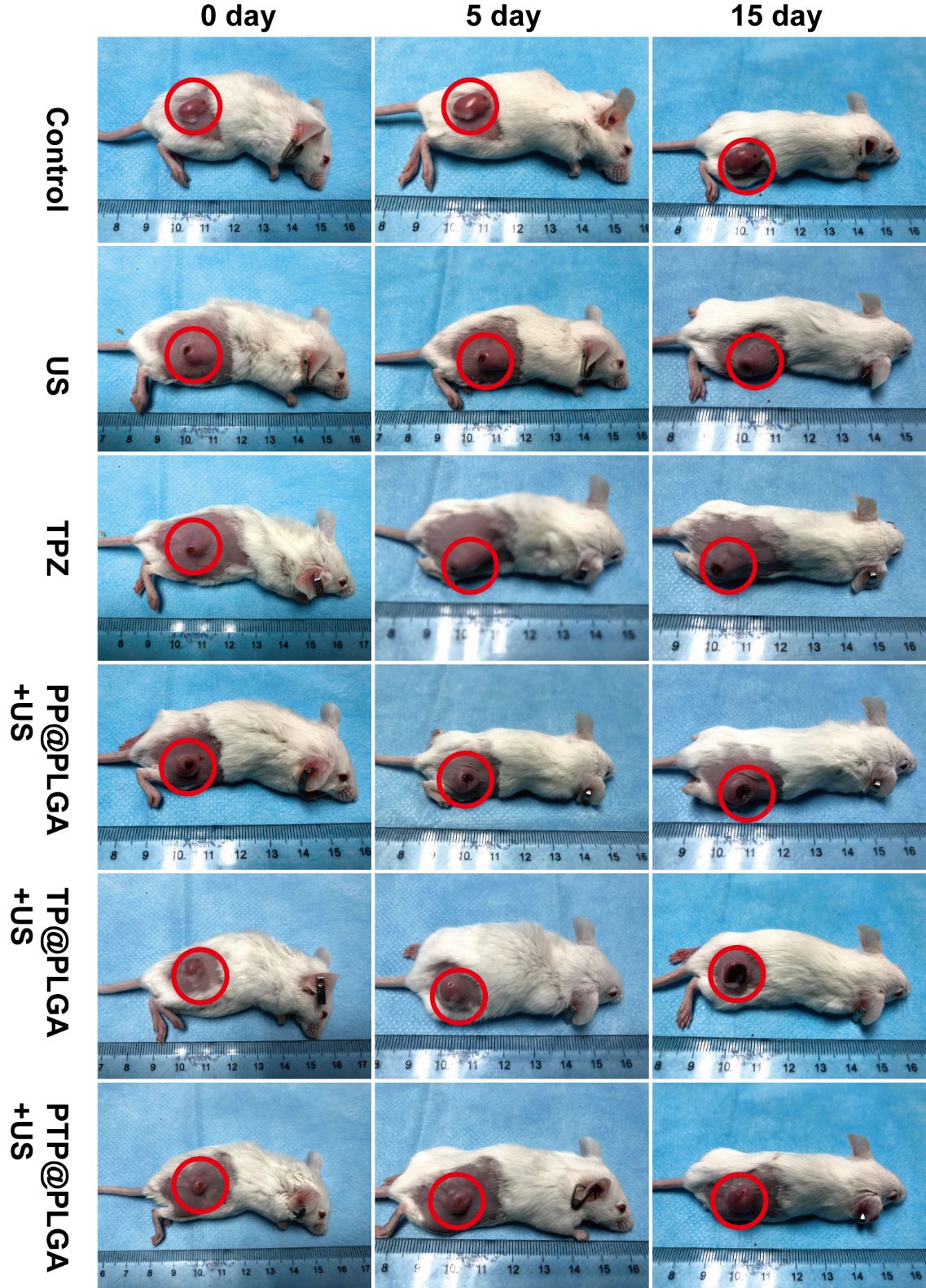


**Figure S10.** Representative photos of tumor bearing mice after different treatments.


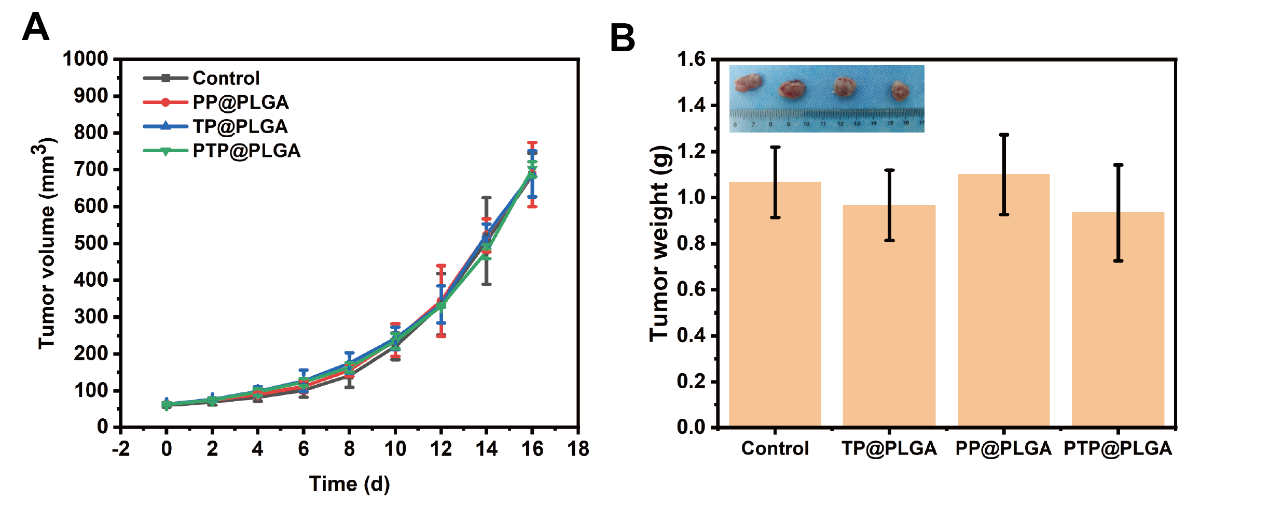


**Figure S11.** (A) Tumor growth profiles of mice in different treating groups. (B) Average weights of tumors collected from different groups 16 days after treatments and their representative images.


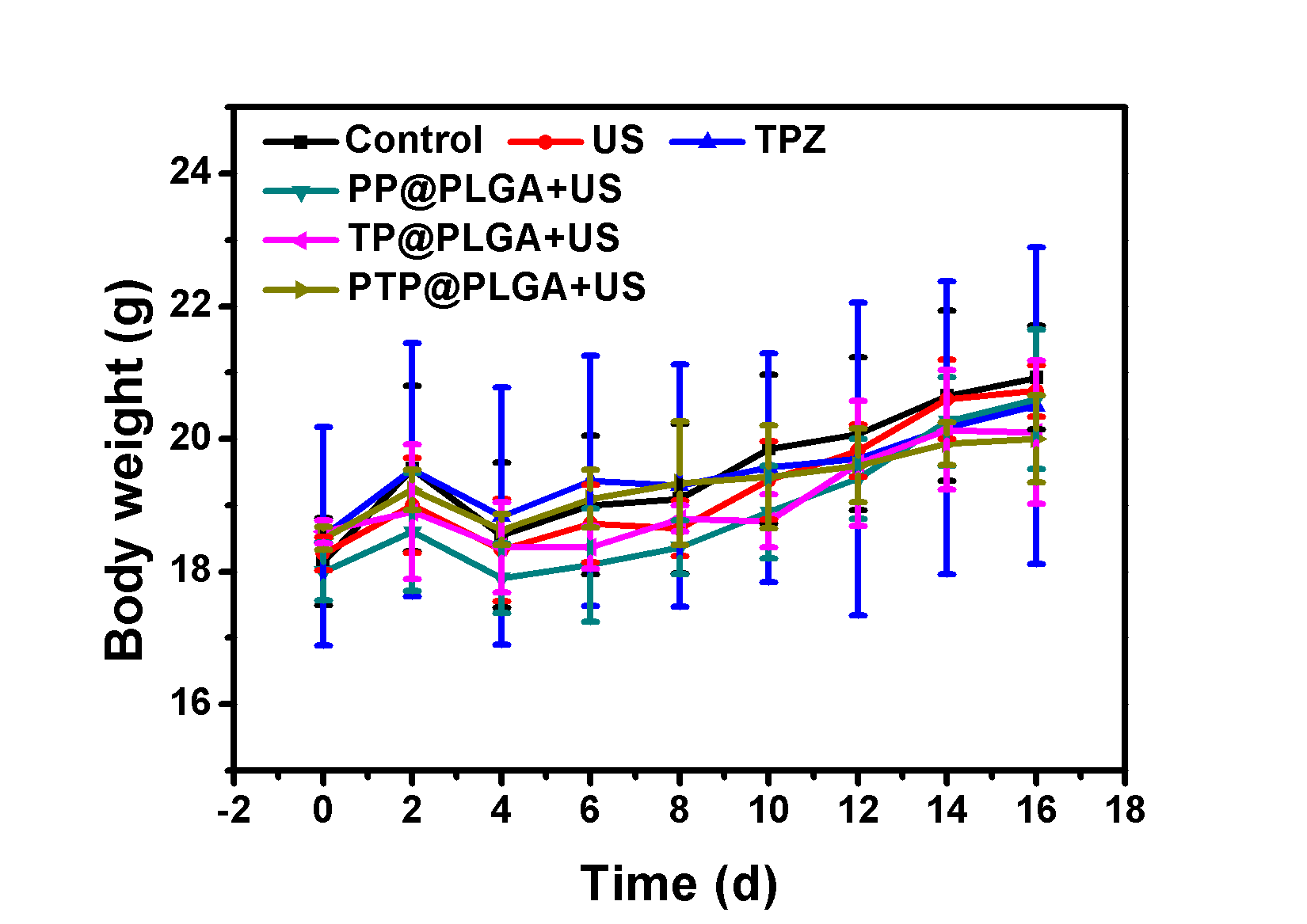


**Figure S12.** The body weight variation of mice in different groups during the treatment.


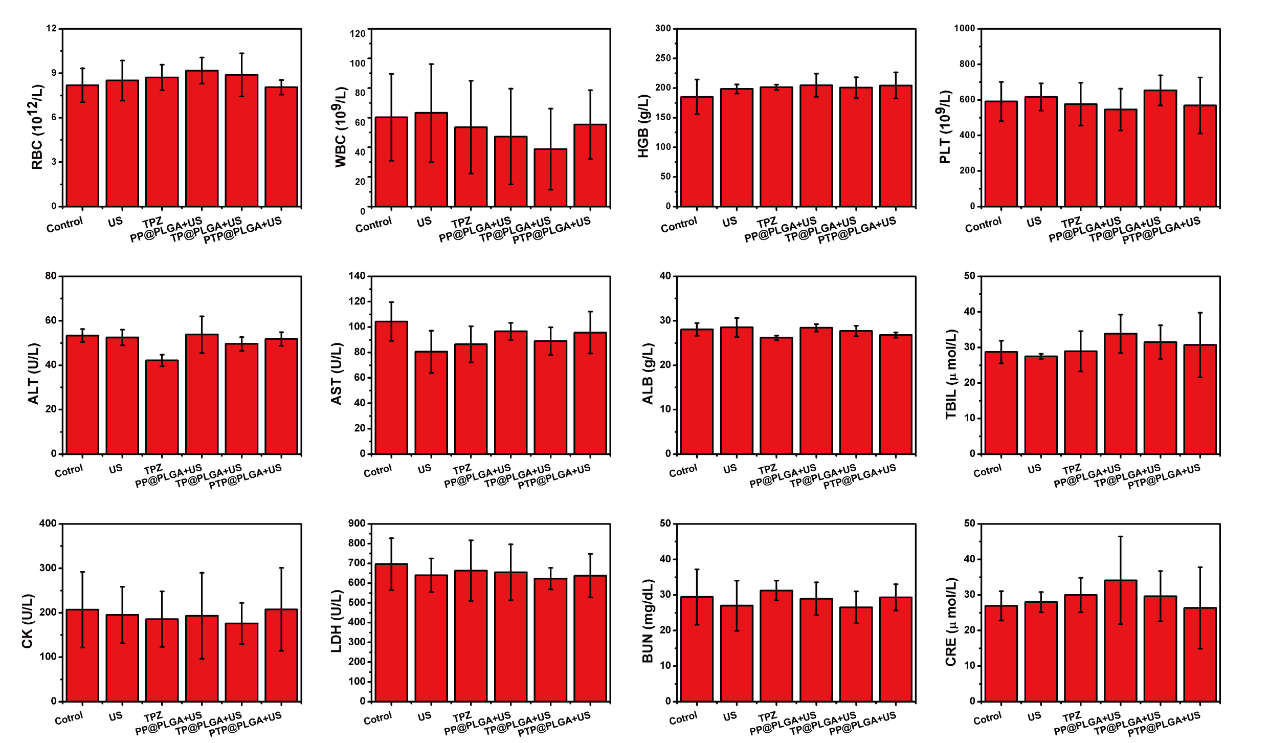


**Figure S13.** Blood biochemical examination of mice at 16th day after receiving different treatments.


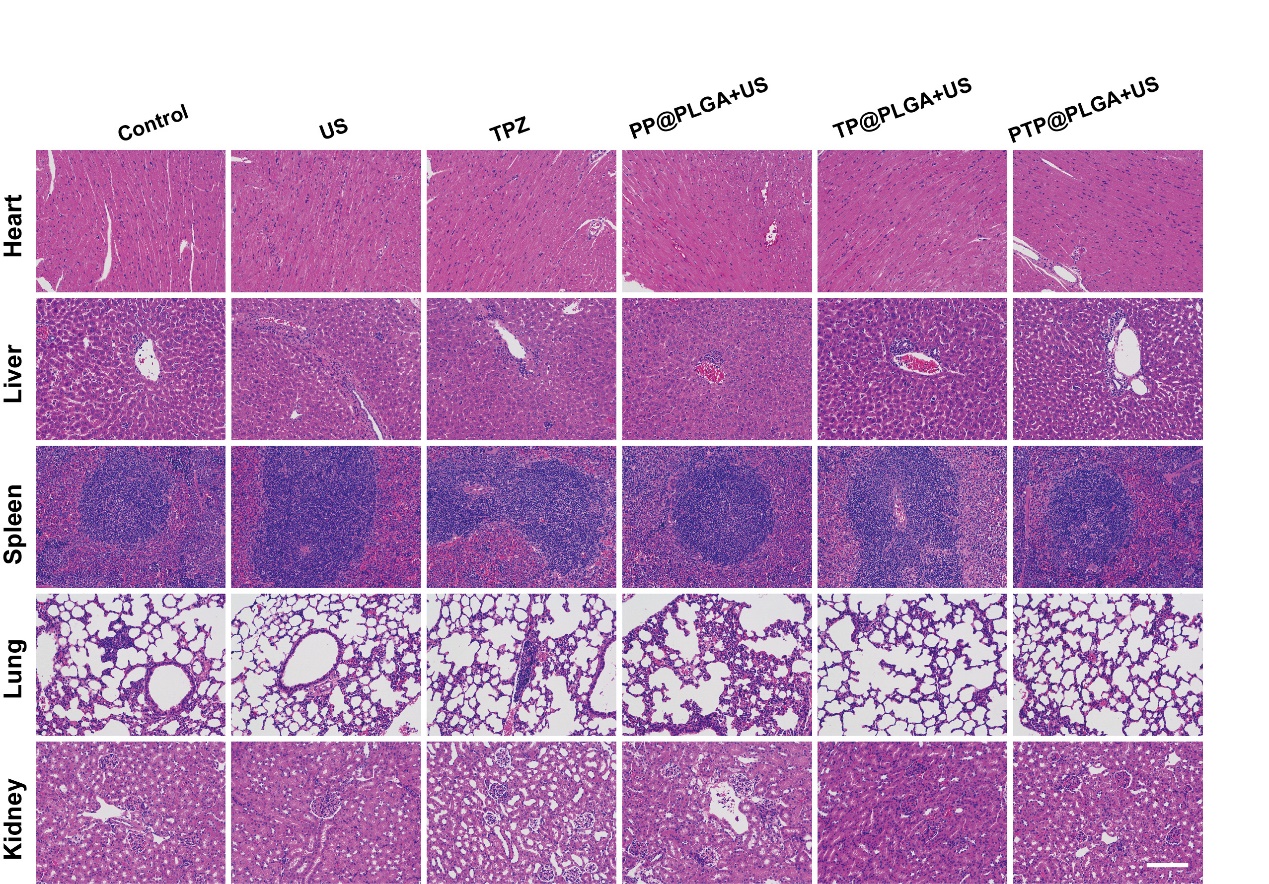


**Figure S14.** H&E staining of major organs at 16th day after mice received different treatments (Scale bar = 20 μm).

**Table S1.** Primer sequences of target genes for PCR

| gene | forward primers (5’-3’) | reverse primers (5’-3’) |
| --- | --- | --- |
| GAPDH | CCTCGTCCCGTAGACAAAATG | TGAGGTCAATGAAGGGGTCGT |
| OCT4 | GAAGAGTATGAGGCTACAGGGACAC | CAGAGCAGTGACGGGAACAGA |
| SOX2 | CTCGCAGACCTACATGAACGG | GCCTCGGACTTGACCACAGA |
| NANOG | TCTACCAGTCCCAAACAAAAGCT | GCCACCGCTTGCACTTCAT |
